# White Spot Syndrome Virus Establishes a Novel IE1/JNK/c-Jun Positive Feedback Loop to Drive Replication

**DOI:** 10.1016/j.isci.2019.100752

**Published:** 2019-11-30

**Authors:** Sheng Wang, Haoyang Li, Shaoping Weng, Chaozheng Li, Jianguo He

**Affiliations:** 1 State Key Laboratory of Biocontrol/ Southern Marine Science and Engineering Guangdong Laboratory (Zhuhai), School of Marine Sciences, Sun Yat-sen University, Guangzhou, 510275, P. R. China; 2 Guangdong Provincial Key Laboratory for Aquatic Economic Animals, School of Life Sciences, Sun Yat-sen University, Guangzhou, 510275, P. R. China; 3 Guangdong Provincial Key Laboratory of Marine Resources and Coastal Engineering, Sun Yat-sen University, Guangzhou 510275, P. R. China

**Keywords:** Biological Sciences, Molecular Biology, Virology, Molecular Microbiology

## Abstract

Viruses need to hijack and manipulate host proteins to guarantee their replication. Herein, we uncovered that the DNA virus white spot syndrome virus (WSSV) established a novel positive feedback loop by hijacking the host JNK pathway via its immediate-early 1 (IE1) protein to drive replication. Specifically, the WSSV IE1 bound to host JNK, and enhanced JNK autoactivation by autophosphorylation, and in turn, elevated JNK kinase activity to its substrate c-Jun and induced *IE1*, which resulted in a viral gene-mediated positive feedback loop. Moreover, the activation of this loop is able to induce *wsv056*, *wsv249*, and *wsv403*, in addition to *IE1* itself. Disruption of this loop during WSSV infection by knockdown of *JNK*, *c-Jun* or *IE1* led to an increased survival rate and lower viral burdens in shrimp. Taken together, this loop may provide a potential target for the development of specific antiviral strategies or agents against WSSV infection.

## Highlights

• Lvc-Jun promotes WSSV *IE1* induction via interacting with the promoter of *IE1* gene
• The interaction of IE1-LvJNK enhances the autophosphorylation of LvJNK
• IE1 hijacks the JNK/c-Jun cascade to create a feedback loop to drive replication
• *wsv056*, *wsv249*, and *wsv403* are also benefit from this positive feedback loop

## Graphical Abstract

**Figure:**
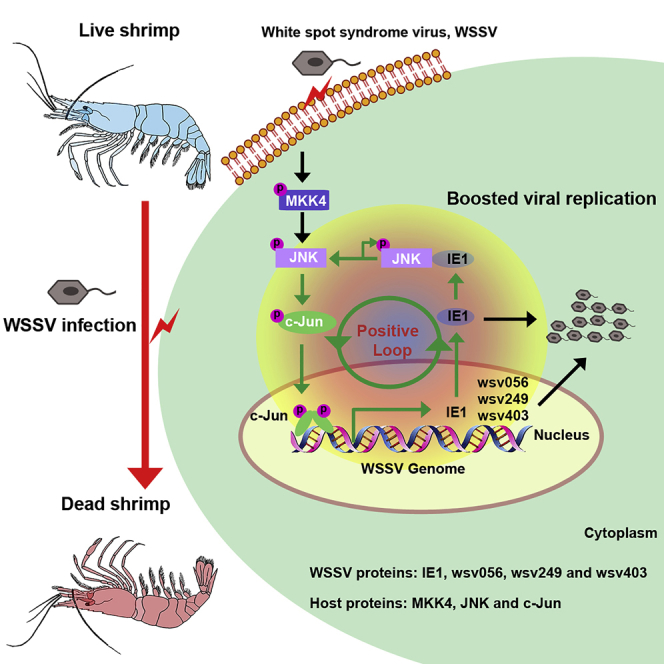

## Introduction

The association between host and virus proteins is crucial for both promoting and suppressing viral replication. Several host signaling pathways as well as host proteins exert significant antiviral effects at multiple steps of viral life cycle. On the contrary, the success of viral infection depends largely on the ability of the virus to manipulate cellular processes through specific interactions with several host factors, which allows the virus to overcome the host immune system and establish a compatible cellular environment for viral replication and proliferation (Hussain and Asgari, 2010, Rana et al., 2013). Moreover, some viruses could even hijack host signaling pathway to promote efficient replication.

Among the studies on the relationship between host signaling pathway and virus replication, mitogen-activated protein kinase (MAPK) pathway is nonnegligible. The MAPK signal transduction pathway is one of the most important immune pathways, and it has been implicated in diverse biological progresses, including proliferation, differentiation, cell survival, apoptosis, and immune responses (Arthur and Ley, 2013, Dong et al., 2002). c-Jun N-terminal kinase (JNK) pathway is one of the three well-known MAPK signaling pathways, which is highly conserved among the fungi, plant, and animal kingdoms (Arthur and Ley, 2013, Li et al., 2011). Considering the importance and conservation of these pathways, they are common targets of both DNA and RNA viruses to generate an intracellular environment suitable for the proliferation and maturation of progeny virions or evade the antiviral immune responses (Arthur and Ley, 2013).

White spot syndrome virus (WSSV), a member of the genus Whispovirus, is a crustacean-infecting, rod-shaped, enveloped, double-stranded circular DNA virus with a genome of approximately 300 kbp (Leu et al., 2009). White spot syndrome (WSS) is the most severe threat to all cultivated species of shrimp worldwide (Siddique et al., 2018). According to the National Shrimp and Crab Industry Technology System, constructed by Ministry of Agriculture of the People's Republic of China, the economic loss caused by WSS is up to $150 million in China every year. As most dsDNA viruses, WSSV immediate-early (IE) genes are the first class of viral genes expressed after primary infection or reactivation, and they are expressed independently of *de novo* viral protein synthesis. They often encode regulatory proteins that are critical controlling downstream viral gene expression and/or modulating the physiological state of the host cell to support viral replication. Thus, IE genes are important in the study of WSSV infection and replication. *IE1*, also named *wsv069*, was one of the 21 identified IE genes (Li et al., 2009, Lin et al., 2011, Liu et al., 2005). Several studies have focused on the role of IE1 in specific interactions between the virus and host proteins. A previous study reported that IE1 interacted with a retinoblastoma (Rb)-like protein in the shrimp *Litopenaeus vannamei* and modulated the cell cycle (G1/S transition) through the Rb-E2F pathway (Ran et al., 2013). Another study also showed that in the shrimp *Penaeus monodon* the thioredoxin protein PmTrx, an important redox regulator, was able to bind to IE1 and restore its DNA-binding activity under oxidizing conditions, indicating a role for IE1 in WSSV pathogenicity (Huang et al., 2012). In addition, WSSV infection was shown to activate several host signaling pathways, and the host transcription factors of these signaling pathways, such as STAT, NF-κB, and AP-1, enhanced the transcription of *IE1* (Huang et al., 2010, Yao et al., 2015, Yao et al., 2016). However, the actual mechanism by which the WSSV regulates the activation of the host immune system for its own replication is unknown.

In this study, we demonstrated that the WSSV *IE1* gene acted as an important positive regulator of the JNK-c-Jun-mediated induction of downstream viral genes, including *IE1*. In particular, WSSV utilized the host JNK pathway to establish a regulatory circuit where IE1 specially interacted with JNK and promoted the autophosphorylation of JNK, which then induced the phosphorylation of c-Jun and activated the *IE1* promoter. This increased *IE1* expression and created a positive feedback loop that further enhanced JNK activity. Such a viral-gene-driven feed-forward mechanism is vital for viral replication, as silencing of either IE1 or JNK resulted in lower viral loads following WSSV infection. These findings provide new insight into how could virus exploit intracellular signaling pathways.

## Results

### Shrimp MKK4-JNK-c-Jun Cascade Was Activated Following WSSV Infection

It is well known that JNK pathways are highly conserved from invertebrates to vertebrates (Li et al., 2011). JNK is typically activated by the phosphorylation of its upstream kinase MKK4/7. Activated JNK then translocates into the nucleus, where it phosphorylates downstream transcription factors, such as c-Jun, thereby modulating cellular transcription (Arthur and Ley, 2013). Previously, some components of the JNK pathway, such as MKK4, JNK, and c-Jun, have been identified in shrimp by different research groups (Li et al., 2015, Shi et al., 2012, Wang et al., 2018, Yao et al., 2015), but there has been minimal research performed establishing a typical MAPK signaling cascade in shrimp. Here, we performed immunoprecipitation and *in vitro* phosphorylation experiments in order to explore whether a MKK4-JNK-c-Jun cascade was conserved in the shrimp *L. vannamei*. As shown in Figure S1A, LvJNK with a V5-tag interacted with both Lvc-Jun-GFP and LvMKK4-GFP but not the control GFP. We next explored the activation cascade of MKK4-JNK-c-Jun by overexpressing pairs of proteins containing either *L. vannamei* MKK4/JNK or JNK/c-Jun in *Drosophila* S2 cells and assessing the phosphorylation of JNK and c-Jun, respectively. The phosphorylation levels of LvJNK (anti-p-JNK) were upregulated significantly with the overexpression of LvMKK4 (Figure S1B), whereas overexpression of LvJNK dramatically induced the phosphorylation levels of Lvc-Jun (anti-p-c-Jun) (Figure S1C). Taken together, these results suggested that the cascade and activation of patterns of MKK4-JNK-c-Jun could be conserved in shrimp *L. vannamei*.

**Figure 1:**
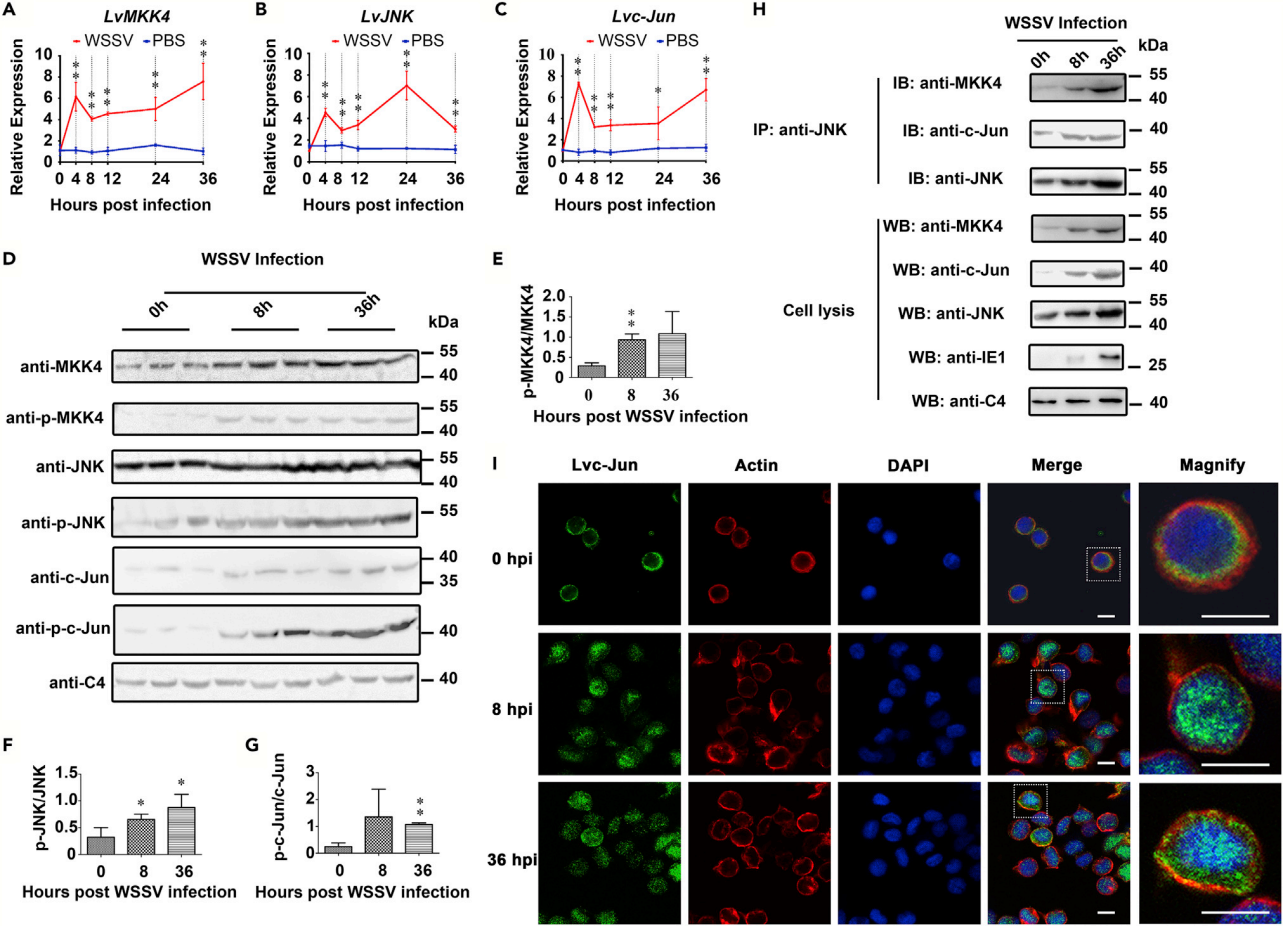
The Shrimp MKK4-JNK-c-Jun Cascade Was Activated by WSSV Infection (A–C) Expression profiles of *LvMKK4* (A), *LvJNK* (B), and *Lvc*-*Jun* (C) after WSSV or PBS (as a control) challenges in hemocytes. Expression levels of genes were assessed by quantitative RT-PCR. (D) The protein expression and phosphorylation levels of LvMKK4, LvJNK, and Lvc-Jun in hemocytes during WSSV infection, which were analyzed by Western blotting using specific antibodies. C4 actin was used as a protein loading control. (E–G) Statistical analysis of phosphorylation ratio of LvMKK4 (E), LvJNK (F), and Lvc-Jun (G) in hemocytes by WCIF ImageJ software corresponding to (D). (H) Increased interaction levels of LvMKK4-LvJNK or LvJNK-Lvc-Jun from 0 to 36 h post-WSSV infection *in vivo*. IE1 antibody was used to confirm the successful infection of WSSV. C4 actin was used as a protein loading control. (I) Lvc-Jun nuclear translocation in response to WSSV infection. Hemocytes were collected at 0, 8, and 36 h post-WSSV infection and then subjected to immunofluorescence staining with a rabbit anti-Jun-specific antibody and mouse anti-β-actin antibody. β-actin was used here in order to define the shape and cytoplasmic region of cells. Immunofluorescence was visualized on a confocal laser scanning microscope. The scale bar = 5 μm. Results (A–C) were expressed as mean ± SD (*n* = 3 independent experiments) and analyzed statistically by Student's *t*-test (**p < 0.01, *p < 0.05). All the experiments were performed three times with similar results. Images were representative of three biological replicates (D, H, and I). See also Figure S1.

Increasing studies have indicated that the JNK pathway can be activated by a number of stressors, including viral infection. In order to investigate whether this pathway in shrimp was involved in some biological processes in response to WSSV invasion, we first addressed the temporal expression patterns of MKK4, JNK, and c-Jun in hemocytes after WSSV challenge using quantitative RT-PCR (qRT-PCR) and Western blotting. The results of qRT-PCR showed that the transcriptional levels of *LvMKK4* (Figure 1A), *LvJNK* (Figure 1B), and *Lvc-Jun* (Figure 1C) were significantly upregulated from 4 to 36 h post-WSSV infection (hpi). Coinciding with the transcriptional levels, Western blotting analysis revealed that the expression and phosphorylation levels of the three proteins were also increased in hemocytes after WSSV challenge (Figure 1D). Gray intensity showed that the phosphorylation ratio of LvMKK4 (Figure 1E), LvJNK (Figure 1F), and Lvc-Jun (Figure 1G) were also upregulated during WSSV infection. As mentioned above, LvMKK4, LvJNK, and Lvc-Jun can form a canonical MKK4-JNK-c-Jun cascade with JNK as middle adaptor *in vitro* (Figure S1). We further checked whether this cascade can signal in response to WSSV challenge *in vivo* through detecting their interactions at three time points (0, 8, and 36 hr) using endogenous immunoprecipitation with specific antibodies. We found that WSSV infection can strongly induce the formation of the MKK4-JNK-c-Jun cascade *in vivo*. In detail, the interactions of LvJNK with both LvMKK4 and Lvc-Jun were clearly detected at 8 and 36 hpi, whereas there was only a weak signal detected at 0 hpi (Figure 1H). Immunofluorescence was performed to probe the nuclear translocation of Lvc-Jun following WSSV infection. As shown in Figure 1I, Lvc-Jun was mainly located within the cytoplasm before infection, but it translocated from the cytoplasm into the nucleus at 8 and 36 hpi *in vivo*. Collectively, these results showed that WSSV challenge induced the activation and phosphorylation of the LvMKK4-LvJNK-Lvc-Jun cascade, as well as the nuclear translocation of Lvc-Jun *in vivo*.

### Activation of the MKK4-JNK-c-Jun Cascade Facilitated WSSV Replication in Shrimp

**Figure 2:**
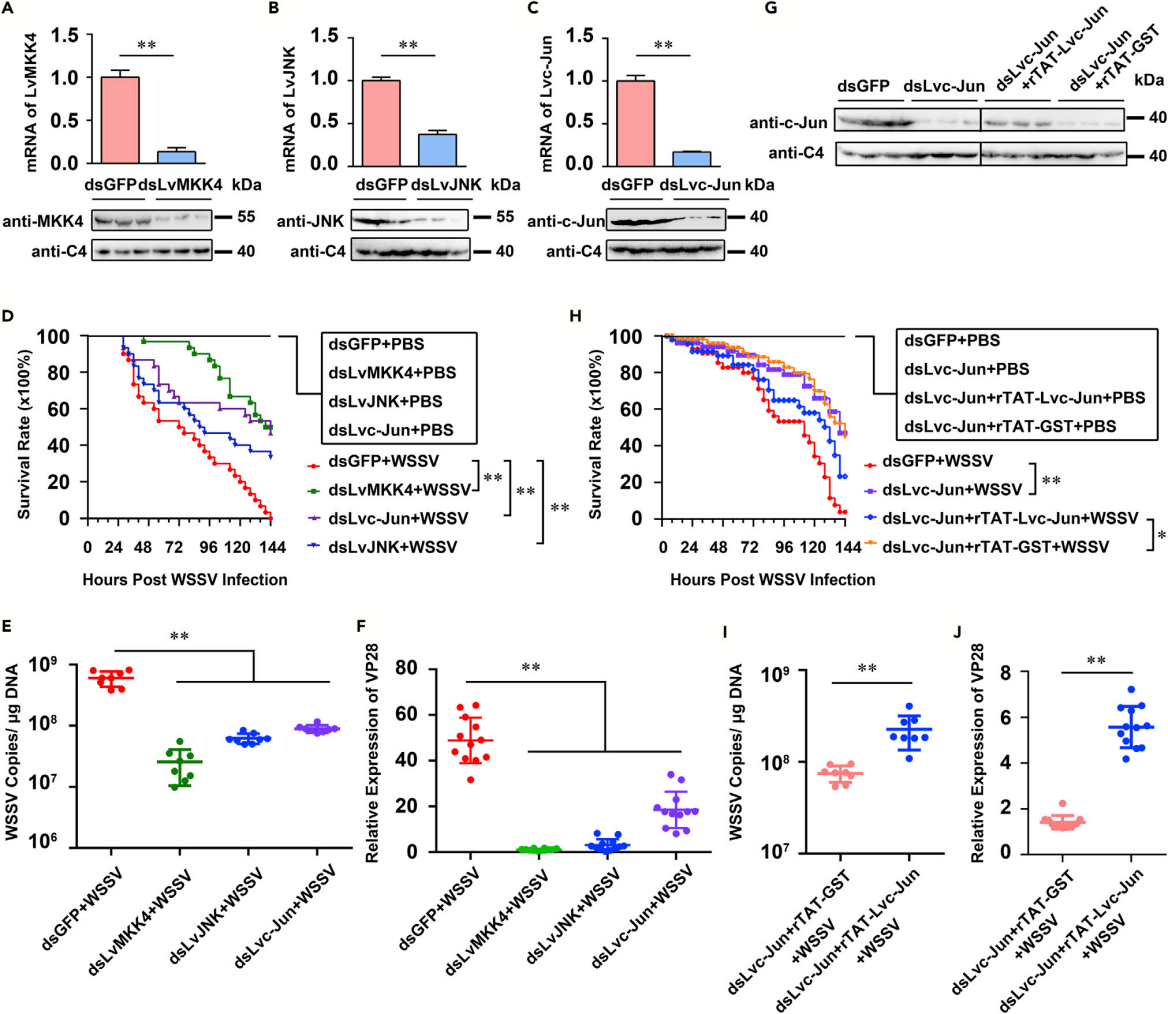
The Shrimp MKK4-JNK-c-Jun Cascade Facilitated WSSV Replication in Shrimp (A–C) Silencing efficiencies of *LvMKK4* (A), *LvJNK* (B), and *Lvc-Jun* (C) in hemocytes. The mRNA levels and protein levels were checked by quantitative RT-PCR (upper panel, mean ± SD, *n* = 3 independent experiments) and Western blotting (lower panel, images were representative of three biological replicates) at 48 h after dsRNA injections, respectively. C4 actin was used as a protein loading control. (D) Silencing of *LvMKK4*, *LvJNK*, or *Lvc-Jun* led to the elevated survival rales in WSSV-infected shrimps, compared with that of the control dsRNA-GFP group. Shrimp survival was monitored every 4 h after WSSV injection. (E and F) Silencing of *LvMKK4*, *LvJNK*, or *Lvc-Jun* enhanced resistance to WSSV infection. The shrimps were injected with WSSV or PBS (as a control) at 48 h after dsRNA injections. The viral loads in the gills were assessed at 48 h post-infection via absolute quantitative PCR (E), and the expression levels of *VP28* in hemocytes were assessed at 48 hpi by quantitative RT-PCR (F). One dot represented 1 shrimp and the horizontal line represented the median of the results. (G) Restoration of Lvc-Jun protein levels in dsRNA-Lvc-Jun-treated shrimp by injection of rTAT-Lvc-Jun protein. Each shrimp was co-injected with dsRNA-Lvc-Jun together with rTAT-Lvc-Jun or with rTAT-GST (as a control). Forty-eight hours later, the protein levels of Lvc-Jun *in vivo* were detected by Western blotting. C4 actin was used as a protein loading control. See also Figures S2 and S4. (H) Rescue of Lvc-Jun protein levels *in vivo* resulted in lower shrimp survival rate during WSSV infection. WSSV and the recombinant proteins were co-injected at 48 h post-*LvMKK4*, -*LvJNK*, or -*Lvc-Jun* silencing, and the death of shrimp was recorded at every 4 h for survival rates analysis. (I and J) Restoration of WSSV replication with injection of recombinant proteins. The copy numbers of WSSV in the gills of shrimp (I) and expression levels of *VP28* in hemocytes (J) in each shrimp were analyzed by absolute quantitative PCR and qRT-PCR, respectively. One dot represented 1 shrimp and the horizontal line represented the median of the results (*n* = 8, E; *n* = 12, F; *n* = 8, I; *n* = 12, J). A Student's *t*-test was applied (**p < 0.01) (A–C, E, F, I, and J). Differences between the groups were analyzed with Log rank test using the software of GraphPad Prism 5.0 (*p < 0.05, and **p < 0.01) (D and H). All the experiments (A–J) were repeated three times with similar results.

Since the MKK4-JNK-c-Jun cascade was activated following WSSV infection in shrimp, we theorized that this activation could either positively or negatively influence WSSV replication. To address this, we performed RNA interference (RNAi) experiments by injecting specific double-stranded RNAs (dsRNAs) against *LvMKK4*, *LvJNK*, and *Lvc-Jun*, followed by WSSV infection. The silencing efficiencies for each gene were assessed by both qRT-PCR and Western blotting (Figures 2A–2C) at 48 h post-dsRNA injection. Our results showed that gene-specific dsRNAs could efficiently silence *LvMKK4*, *LvJNK,* and *Lvc*-*Jun* at the transcriptional levels in hemocytes (Figures 2A–2C, upper panels). Consistent with the mRNA levels, Western blotting showed that the protein levels of LvMKK4, LvJNK, and Lvc-Jun were also significantly suppressed by dsRNA-LvMKK4, dsRNA-LvJNK, and dsRNA-Lvc-Jun, respectively (Figures 2A–2C, down panels). Next, we challenged RNAi-treated shrimp with WSSV or PBS (as mock) at 48 h post-dsRNA injection and recorded the number of shrimps that died every 4 h. As shown in Figure 2D, compared with the GFP dsRNA control group (0%), the survival rates were significantly increased in the LvMKK4-dsRNA-treated group (∼50%, p < 0. 01), LvJNK dsRNA-treated group (∼33.3%, p = 0.0079 < 0.01), and Lvc-Jun-dsRNA-treated group (∼46.7%, p < 0. 01) after WSSV infection. In addition, there was no mortality observed with PBS challenge alone, indicating that each dsRNA injection itself did not result in shrimp death. To further investigate whether the increased survival rates of shrimp injected with dsRNA-LvMKK4, dsRNA-LvJNK, or dsRNA-Lvc-Jun were due to the increased resistance to WSSV or decreased replication of WSSV, we next analyzed the viral titers in shrimps by absolute quantitative PCR (aq-PCR) and *VP28* transcription levels (a viral late, structural protein) by qRT-PCR. In accordance with the results of survival rates, we observed that both the WSSV copy number (Figure 2E) and *VP28* expression levels (Figure 2F) were markedly decreased in dsRNA-LvMKK4, dsRNA-LvJNK, and dsRNA-Lvc-Jun groups compared with those of the control dsRNA-GFP group. These data strongly indicated that the LvMKK4-LvJNK-Lvc-Jun cascade in shrimp was critical for WSSV replication. We reasoned that the lessened viral loads or the survival rate phenotype in dsRNA-injected shrimps could be rescued by co-injection with exogenous recombinant protein. To further validate this, we treated shrimp by co-injection of Lvc-Jun-dsRNA and an affinity-purified rTAT-Lvc-Jun protein or rTAT-GST tag (Figure S2) as a control, and 48 h later the shrimp were infected with WSSV. Injection of dsRNA-Lvc-Jun significantly suppressed the total protein levels of Lvc-Jun in hemocytes, whereas the purified rTAT-Lvc-Jun protein compensated, to some extent, for the reduced protein level of Lvc-Jun caused by *in vivo* RNAi (Figure 2G). We found that injection of rTAT-Lvc-Jun contributed to the lower survival rate (Figure 2H), as well as enhanced the WSSV copy number (Figure 2I) and *VP28* expression levels (Figure 2J) in shrimp during WSSV infection, compared with those of control group. Taken together, these results convincingly demonstrated that the activated LvMKK4-LvJNK-Lvc-Jun cascade facilitated WSSV replication after infection *in vivo*.

### c-Jun Was Hijacked by WSSV to Promote IE1 Expression in Shrimp

**Figure 3:**
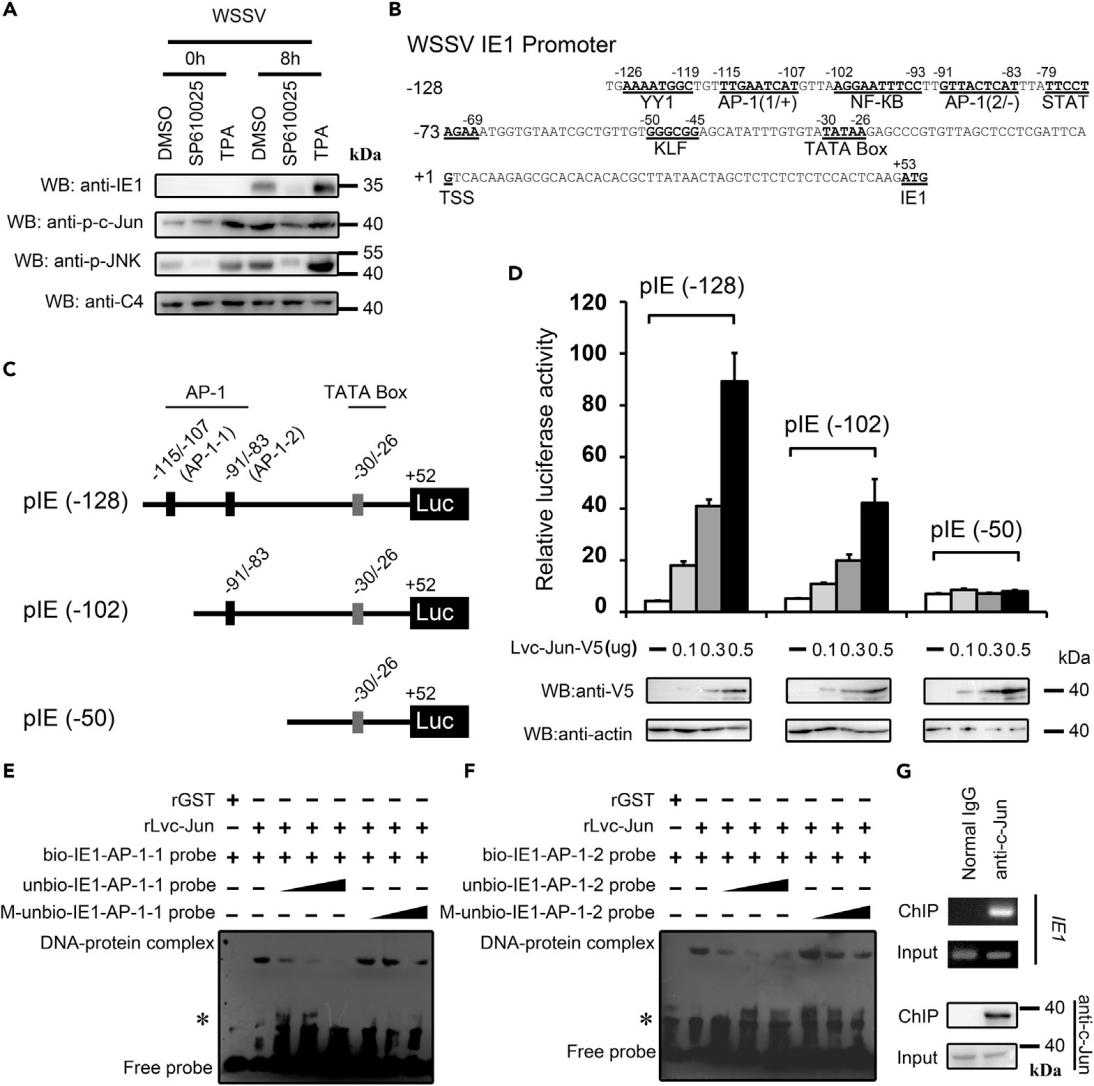
Shrimp c-Jun Was Involved in Regulating *IE1* Expression *In Vitro* and *In Vivo* (A) The shrimp JNK-c-Jun cascade is involved in regulating the expression of WSSV IE1 *in vivo*. The protein expression of WSSV IE1 was significantly downregulated by the treatment with inhibitor SP600125, whereas its expression was evidently upregulated by the treatment with activator TPA. C4 actin was used as a protein loading control. (B) The structure of the promoter of WSSV *IE1*. There were several putative transcription factors binding motifs in the promoter of *IE1*, including two AP-1 binding motifs located at −115 to −107 (named as AP-1-1) and −91 to −83 (named as AP-1-2). (C) Description of IE1 promoter-truncated mutants used in the dual reporter genes assay. The pIE (−128) contained two AP-1 binding motifs (upper panel), pIE (−102) contained one AP-1 binding motif (middle panel), and pIE (−50) had both of AP-1 binding motifs deleted (lower panel). (D) Effects of Lvc-Jun overexpression on the promoter activities of *IE1 in vitro*. Ectopic expression of Lvc-Jun in *Drosophila* S2 cells was able to upregulate the promoter activities of pIE (−128) and pIE (−102), but not the pIE (−50), in a dose-dependent manner. C4 actin was used as a protein loading control. (E and F) Lvc-Jun interacted with the AP-1 binding sites from the *IE1* promoter *in vitro* by EMSA assay. Combination of rLvc-Jun proteins with the AP-1-1 site (E) and AP-1-2 site (F). See also Figure S3. (G) WSSV infection induced Lvc-Jun binding to the promoter region of *IE1 in vivo*. ChIP assays were performed with shrimp hemocytes at 24 h post-WSSV infection. Semi-quantitative PCR was used to check the interaction of Lvc-Jun with the promoter region of WSSV *IE1* in anti-c-Jun ChIP sample and normal rabbit IgG ChIP sample. The bars (D) indicated the mean ± SD of the luciferase activities (*n* = 6). Images were representative of three biological replicates (A and D–G).

We wanted to explore the mechanism that the WSSV employs to hijack the LvMKK4-LvJNK-Lvc-Jun cascade for its own needs, including viral gene expression and genome replication. As observed above, the LvMKK4-LvJNK-Lvc-Jun cascade was activated at a very early stage of WSSV infection, as illustrated by the increased phosphorylation ratio of LvJNK and Lvc-Jun at 8 hpi (Figures 1D, 1F, and 1G). c-Jun is a transcription factor of the AP-1 family, and the transcription of WSSV *IE* genes, which are independent of *de novo* viral protein synthesis, are often driven directly by host transcription factors (Huang et al., 2010, Qiu et al., 2014, Yao et al., 2016). Thus, it is easy to speculate that Lvc-Jun could be hijacked by the WSSV to promote expression of its own genes, such as *IE1*. To address this, we analyzed the expression level of IE1 during WSSV infection (at 8 hpi) under conditions that this pathway was inhibited or activated by treatment with a JNK pathway inhibitor SP600125 or activator 12-O-tetradecanoylphorbol-3-acetate (TPA), respectively. We observed that the phosphorylation levels of both LvJNK (anti-p-JNK) and Lvc-Jun (anti-p-c-Jun) were efficiently inhibited or activated by SP600125 or TPA *in vivo*, respectively (Figure 3A). As expected, the expression levels of IE1 during WSSV infection were depressed and upregulated with treatments of SP600125 and TPA *in vivo*, respectively, which were in line with the phosphorylation levels of LvJNK and Lvc-Jun, respectively (Figure 3A). These results strongly indicated that WSSV could utilize the transcriptional regulator of c-Jun to stimulate the expression of *IE1*. We next found two potential AP-1 binding motifs present in the promoter region of WSSV *IE1*, located at approximately −115 through −107 (AP-1-1) and −91 through −83 (AP-1-2) from the transcriptional start site (TSS) (Figure 3B). To test whether Lvc-Jun could induce IE1 expression via the two putative AP-1 binding motifs, we constructed three reporter plasmids that contained truncated promoter regions of *IE1* with two or one or no AP-1 motifs, named pIE (−128), pIE (−102), and pIE (−50), respectively (Figure 3C). A dual-luciferase reporter assay showed that both pIE (−128) and pIE (−102) could be upregulated by ectopic expression of Lvc-Jun in *Drosophila* S2 cells in a dose-dependent manner (Figure 3D), suggesting that both putative AP-1 binding motifs play important roles in the Lvc-Jun-mediated *IE1* induction. To confirm this, EMSA experiments were performed to investigate the direct interaction between Lvc-Jun and AP-1 motifs. Recombination GST-tagged Lvc-Jun protein (rLvc-Jun-GST) was expressed in *Escherichia coli*, purified, and the GST tag was removed to obtain rLvc-Jun (Figure S3). Our results showed that band shifts of protein-DNA complexes were detected when rLvc-Jun proteins were incubated with biotin-labeled IE1-AP-1-1 probe (Figure 3E, lane 2). In addition, the band shifts could be competitively reduced when rLvc-Jun proteins were incubated with wild-type unbiotinylated IE1-AP-1-1 probes at a 10-, 50-, and 100-fold molar excess (Figure 3E, lane 3–5) but not the mutant unbiotinylated IE1-AP-1-1 probes at a 10-, 50-, and 100-fold molar excess (Figure 3E, lane 6–8). A similar result was observed in the interaction between rLvc-Jun protein and the IE1-AP-1-2 motif (Figure 3F). In the control sets (rGST proteins), no band shift was observed, which suggests that the interaction between rLvc-Jun protein and each of the AP-1 binding motif is specific. We further explored whether endogenous Lvc-Jun could be able to bind to the promoter of *IE1 in vivo*. The hemocytes were isolated from shrimp at 24 h post-WSSV infection. WSSV-infected hemocytes were subjected to ChIP assays with an anti-c-Jun antibody or normal rabbit IgG (as control). Figure 3G showed that the promoter region of *IE1* containing the AP-1 binding motif could be visibly detected by semi-quantitative PCR in the anti-c-Jun antibody ChIP sample, whereas no amplification signal could be detected in IgG ChIP sample. Collectively, these *in vivo* and *in vitro* results strongly demonstrated that Lvc-Jun could be hijacked by the WSSV to promote *IE1* gene expression.

### IE1 of the WSSV Interacted with Shrimp JNK to Generate a Positive Feedback Loop

**Figure 4:**
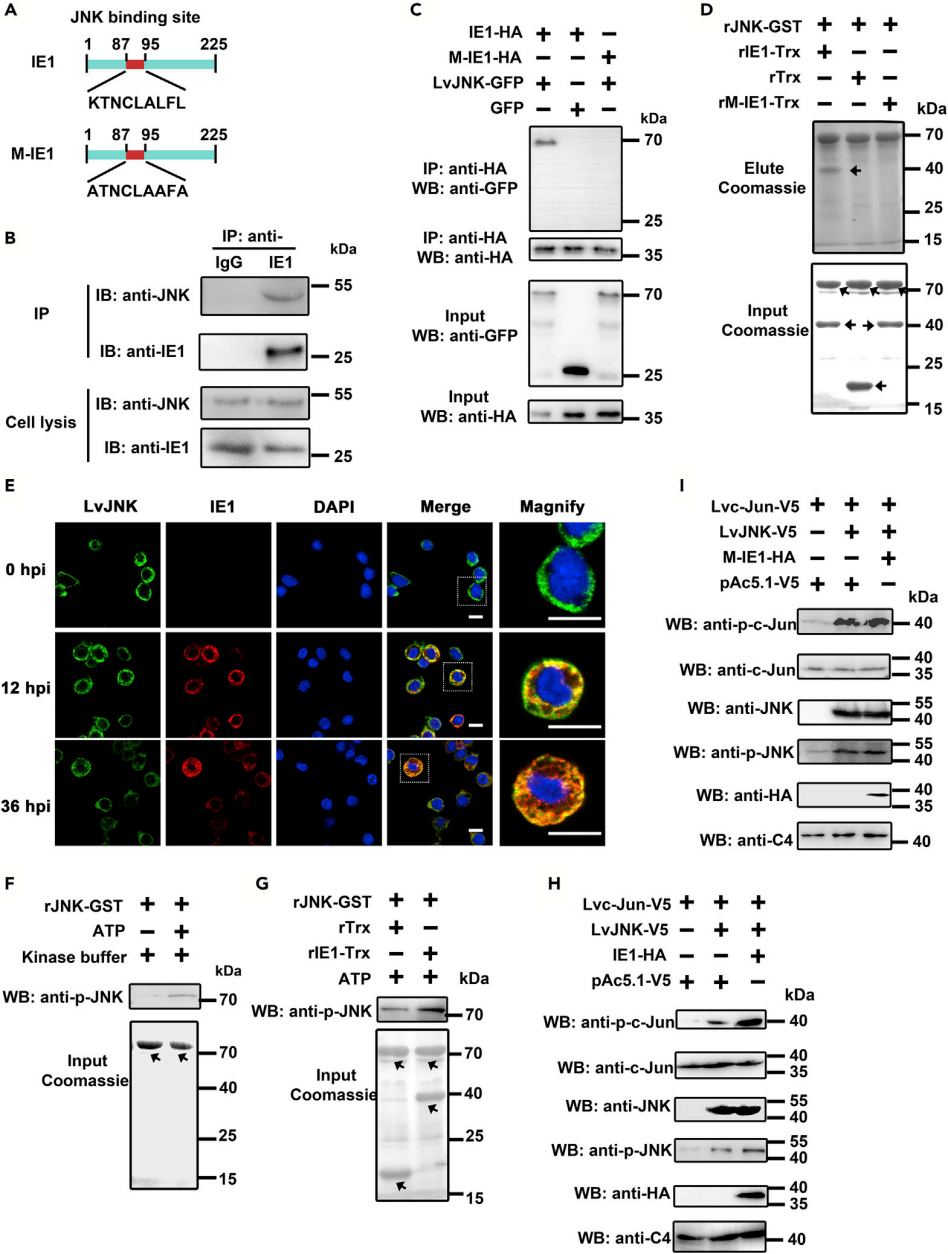
The IE1 of WSSV Combined with Shrimp JNK, Forming a Positive Feedback Loop (A) Description of the IE1-site mutant used in this study. The wild-type of IE1 contained a consensus sequence of ^87^KTNCLALFL^95^ for JNK-binding domain (JBD). The crucial amino acid residues K^87^, L^93^, and L^95^ from JBD of IE1 were mutated to A^87^, A^93^, and A^95^ to generate mutant IE1 (M-IE1). (B) Endogenous IP showed that WSSV IE1 interacted with LvJNK in hemocytes (*in vivo*) at 24 h postinfection. (C) Co-IP analysis showed the IE1, but not the M-IE1, interacted with LvJNK. (D) GST pulldown further confirmed the IE1-LvJNK interaction via the JNK-binding motif. (E) Immunofluorescence experiments showed that LvJNK colocalized with IE1 in hemocytes (*in vivo*) at 12 h and 36 h post-WSSV infection in shrimp. The scale bar = 5 *μ*m. (F) LvJNK underwent autophosphorylation in the ATP and kinase buffer *in vitro*. Identical protein inputs were checked by SDS-PAGE with Coomassie staining. (G) IE1 was able to enhance the autophosphorylation activity of LvJNK in a kinase buffer *in vitro*. Identically quantitative protein inputs were checked by SDS-PAGE with Coomassie staining. (H and I) Co-expression of IE1 (H), but not the M-IE1 (I), and LvJNK led to the higher phosphorylation levels of both LvJNK and its substrate Lvc-Jun *in vitro*. C4 actin was used as a protein loading control. Images were representative of three biological replicates (B–I). See also Figures S4 and S5.

The above results showed that the activated LvJNK pathway was utilized by the WSSV for *IE1* expression. In fact, the LvJNK-Lvc-Jun cascade was activated throughout WSSV infection, as indicated by the continually increased phosphorylation levels of both LvJNK and Lvc-Jun (Figures 1D, 1F, and 1G). However, it was unknown how the WSSV achieved this. Thus, we speculated that the WSSV employed a strategy that potentially directly targeted intracellular signaling molecules of the host in order to maintain a sustained activation of the LvJNK-Lvc-Jun cascade. The MAPKs, including JNK, are commonly targeted by many viral genes to manipulate cellular processes for their benefit. IE1, regulated by the LvJNK-Lvc-Jun cascade, was a top candidate to search for motifs interacting with JNK. We found that IE1 contained a consensus sequence of ^87^KTNCLALFL^95^ for a JNK-binding domain (JBD), which followed the pattern R/K_1-3_-X_1-6_-L/I-X-L/I (Ho et al., 2003). We performed both an endogenous immunoprecipitation (*in vivo*) and pulldown assay (*in vitro*) to assess the potential interaction between IE1 and LvJNK. Hemocytes from WSSV-infected shrimp were collected for endogenous immunoprecipitation with a specific IE1 antibody and normal rabbit IgG as a control, followed by Western blot analysis using anti-JNK antibody. The results showed that LvJNK interacted with IE1 at 24 h after WSSV infection *in vivo* (Figure 4B). In the pulldown assay, we constructed plasmids expressing wild-type IE1 and M-IE1, which had the putative JNK-binding site mutated by replacing K87, L93, and L95 with A87, A93, and A95 (Figures 4A and S4). Both Co-IP (Figure 4C) and pulldown (Figure 4D) experiments showed that LvJNK could interact with IE1, but not M-IE1, suggesting that IE1 could interact with LvJNK through the JNK-binding site. In addition, immunofluorescence experiments in hemocytes showed that endogenous LvJNK (green fluorescence) partially colocalized with IE1 (red fluorescence), which merged into yellow at 12 or 36 h post-WSSV infection (Figure 4E). Taken together, our results demonstrated that IE1 interacted with LvJNK via its JNK-binding site.

Next, in order to explore the effects of the IE1-LvJNK interaction on the activity of LvJNK, especially if the phosphorylation levels of LvJNK varied, we carried out a phosphorylation assay with expressed and purified GST*-*tagged LvJNK from *E. coli*. We first observed that rLvJNK-GST could phosphorylate itself in a phosphorylation environment *in vitro* (Figure 4F), which led us to hypothesize that the IE1-LvJNK interaction could play an important role in the autophosphorylation of JNK. As shown in Figure 4G, we found that the phosphorylation level of rLvJNK was higher with rIE1-Trx, as compared with that of the control group. These results demonstrated that LvJNK could undergo autophosphorylation and that IE1 had the ability to enhance the autophosphorylation of LvJNK *in vitro*. To further verify whether their interaction is required for the LvJNK autophosphorylation enhancement, and whether this outcome can promote LvJNK to phosphorylate its downstream substrate Lvc-Jun, we performed protein kinase assays in *Drosophila* S2. The results showed that co-expression of IE1 and LvJNK led to higher phosphorylation levels of both LvJNK and its substrate Lvc-Jun (Figure 4H), whereas the mutated IE1 had lost its ability to achieve this (Figure 4I). In mammals, JNK2α2 molecule had the ability to bind each other for autophosphorylation. The autoactivation of JNK2α2 followed a “binding each other first, phosphorylation second” manner (Cui et al., 2005). We also found that overexpression of IE1 increased LvJNK-LvJNK combination *in vitro* (Figure S5). It may be the mechanism by which IE1 enhanced the autophosphorylation of LvJNK and is worthy of further investigation. In combination with the observations above, we concluded that IE1 could hijack this cascade by targeting LvJNK to generate a positive feedback loop. Such a scheme is the viral-gene (IE1)-driven amplification loop, in which WSSV infection (pathogenic stress) induced the MKK4-JNK-c-Jun cascade, activated the IE1 gene promoter, and increased IE1 expression feedback to enhance JNK activity.

### Disruption of the IE1/JNK/c-Jun Positive Feedback Loop Suppressed the Pathogenesis of the WSSV

**Figure 5:**
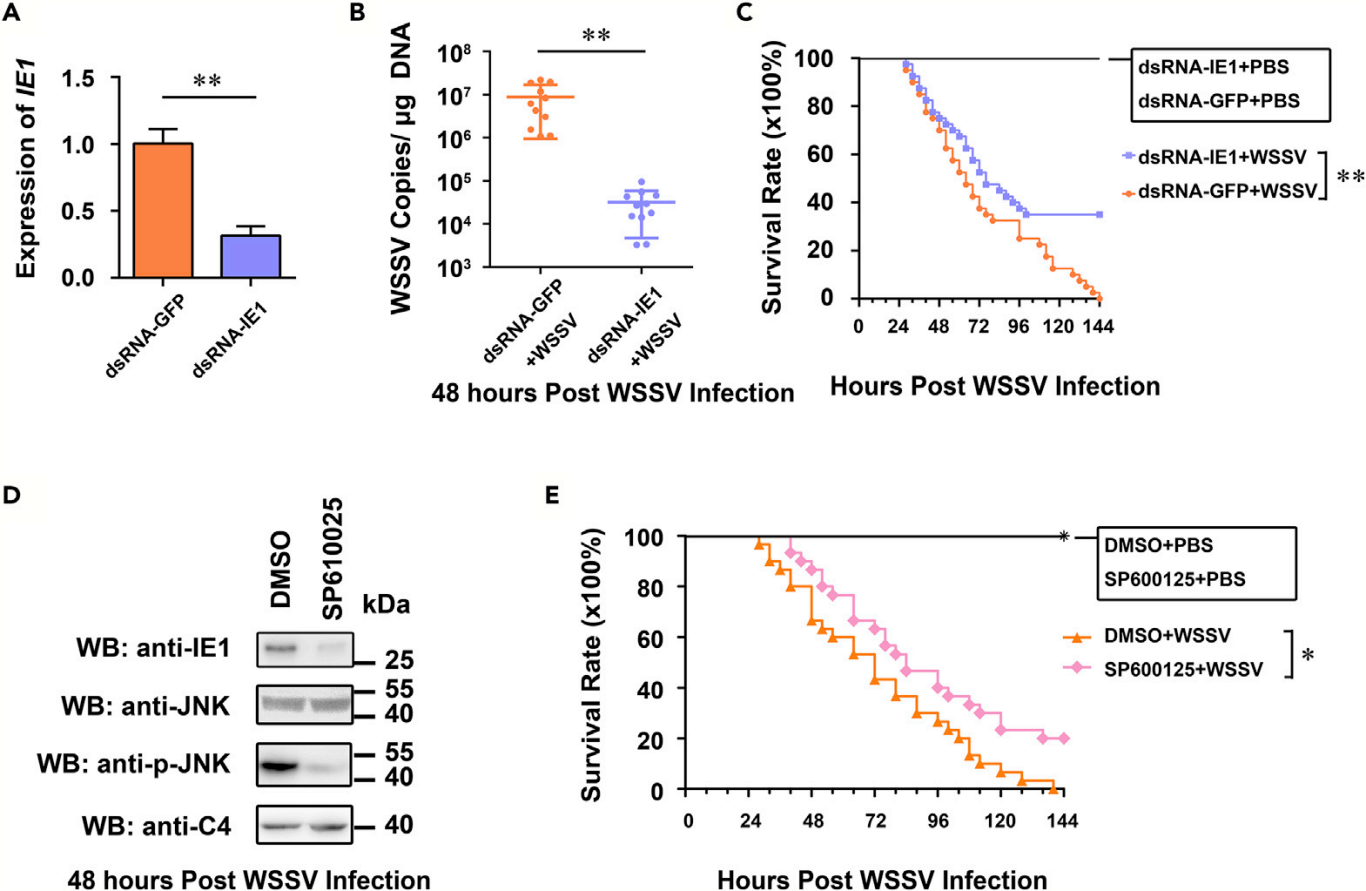
IE1-Driven IE1/JNK/c-Jun Positive Feedback Loop Was Vital for the Pathogenesis of the WSSV (A) Knockdown efficiencies of *IE1* was confirmed by quantitative RT-PCR. (B) Silencing of *IE1* reduced WSSV replication in shrimps. The viral loads in the gills of shrimp were assessed at 48 hpi via absolute quantitative PCR. (C) Survival of WSSV-challenged *IE1*-silenced shrimp and GFP dsRNA-treated shrimp. (D) SP600125 inhibited LvJNK phosphorylation during WSSV infection. (E) Survival of WSSV-challenged SP600125-treated shrimps and DMSO-treated shrimps. A Student's *t*-test was applied (**p < 0.01) (A and B). One dot represented 1 shrimp and the horizontal line represented the median of the results (*n* = 11, B). Differences between the groups were analyzed with Log rank test using the software of GraphPad Prism 5.0 (**p < 0.01) (C and E). The experiment was repeated two or three times with similar results.

Because IE1 of the WSSV is the indispensable constituent to form the positive feedback loop, we performed targeted dsRNA treatments to assess the functional importance of the IE1 for WSSV pathogenesis. IE1 dsRNAs were co-injected with WSSV to knockdown *IE1* expression during WSSV infection. Efficient silencing of *IE1* mRNA was observed at 48 h post-dsRNA injection by qRT-PCR (Figure 5A). We found that silencing of *IE1* led to lower viral burdens in shrimp at 48 h post-WSSV infection, compared with those of GFP-dsRNA-inoculated shrimps (Figure 5B). In addition, in a parallel survival experiment, we observed that *IE1* dsRNA-treated shrimps died more slowly and had an increased survival rate (χ^2^: 10.00, p = 0.0016 < 0.01), compared with those of dsRNA-GFP group (Figure 5C). Additionally, SP600125 was also used *in vivo* to verify the importance of JNK-c-Jun activation in WSSV infection. Shrimps were injected with SP600125 or DMSO (as a control), followed by WSSV injection. The inhibitory efficiency of SP600125 was detected at 48 h post-WSSV infection (Figure 5D). Results showed that SP600125 could suppress the expression of IE1, as well as the phosphorylation level of LvJNK, but not the protein levels of LvJNK (Figure 5D). Moreover, in a parallel survival experiment, we observed that SP600125-treated shrimps began to die 12 h later and had an increased survival rate (χ^2^: 5.659, p = 0.0174 < 0.05), compared with those of the DMSO-treated control group (Figure 5E). Therefore, these results demonstrated that the positive feedback loop induced by and resulting in IE1 accumulation via JNK-c-Jun activation could facilitate WSSV pathogenesis, including viral replication and viral pathogenicity.

### The IE1-JNK-c-Jun Loop Regulated Other WSSV *IE* Genes *In Vitro* and *In Vivo*

**Figure 6:**
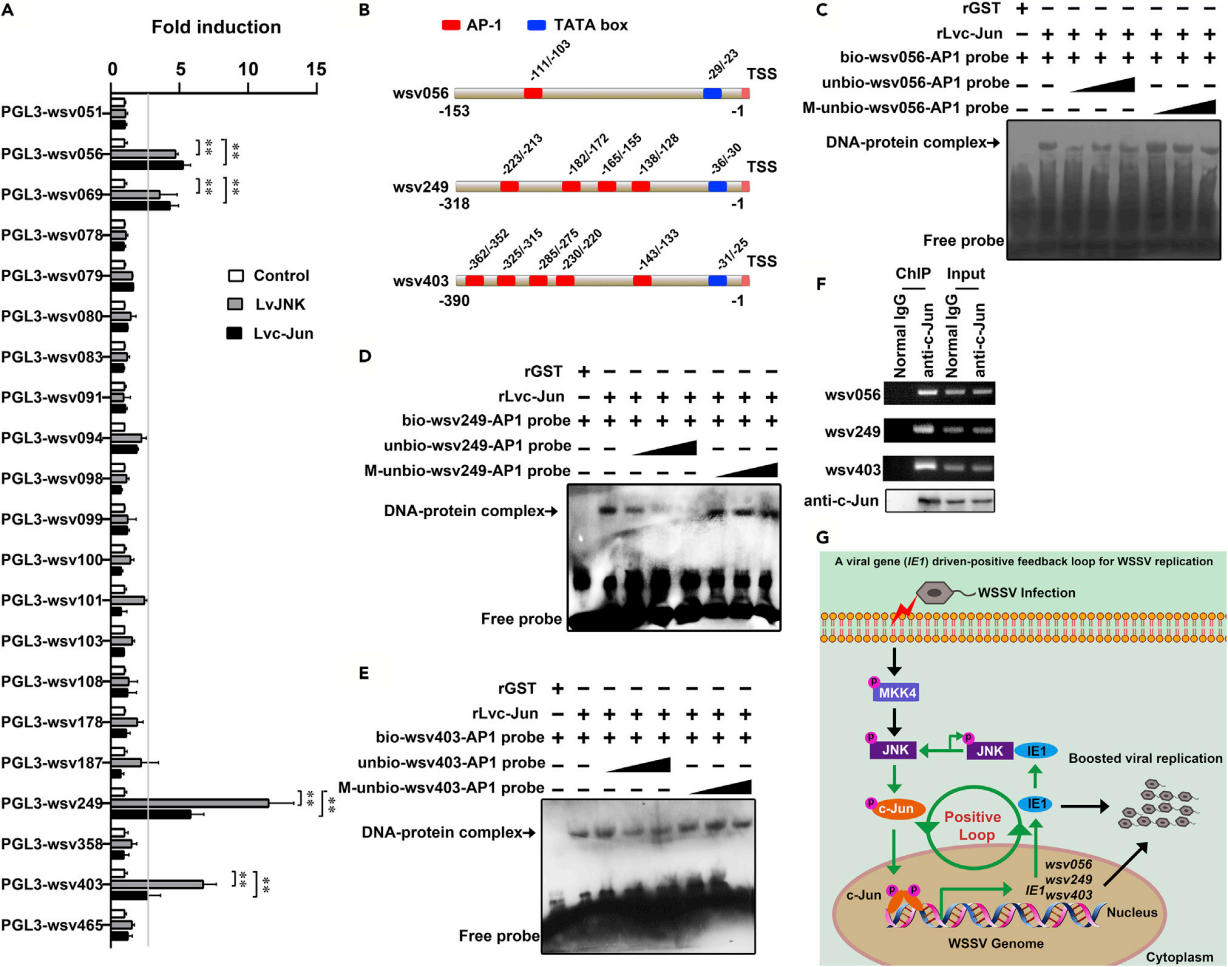
The IE1 Driven-Positive Feedback Loop Regulated the Expression of Other WSSV *IEs* (A) Dual reporter assay screened the effects of LvJNK or Lvc-Jun expression on the promoter activities of all 21 WSSV *IE* in *Drosophila* S2 cells. The horizontal line indicated a 3-fold induction threshold. (B) The potential AP-1 binding sites in the promoter regions of *wsv056*, *wsv249,* and *wsv403*. The putative AP-1 binding motifs were presented as red rectangles, whereas TATA boxes were presented as blue rectangles. We chose the AP-1 binding motifs (asterisk noted) that were closest to the transcriptional start site (TSS) of *wsv056*, *wsv249,* and *wsv403* for following EMSA assay. (C–E) EMSA assay showed that rLvc-Jun bound with a putative AP-1 binding motif from promoters of *wsv056* (C), *wsv249* (D)*,* or *wsv403* (E). (F) ChIP assays confirmed that the Lvc-Jun interacted with the promoter regions of *wsv056*, *wsv249,* and *wsv403 in vivo*. (G) Model of the IE1-driven positive feedback loop for WSSV replication. WSSV infection (pathogenic stress) induced an initial activation of the LvMKK4-LvJNK-Lvc-Jun cascade, which triggered WSSV immediate-early gene *IE1* expression. The IE1 protein then feedbacked to interact with JNK and promote its autophosphorylation, and in turn, triggered the downstream c-Jun (phosphorylation and translocated to nuclear) to induce the transcription of *IE1 itself*, which created a positive feedback loop. The IE1-driven positive feedback loop kept sustained activation of LvJNK-Lvc-Jun cascade, which further resulted in the expression of other *IE* genes such as *wsv056*, *wsv249*, and *wsv403*, in addition to *IE1* (*wsv069*), and finally facilitated WSSV replication. The bars indicated the mean ± SD of the luciferase activities (*n* = 6, A). A Student's (A) *t* test was applied (**p < 0.01). Images were representative of three biological replicates (A and C–F). See also Figure S3.

In addition to *IE1*, we were curious if other *IEs* were also induced by the IE1-LvJNK-Lvc-Jun loop. To assess this, all 21 WSSV *IEs* were screened through overexpression of LvJNK or Lvc-Jun in *Drosophila* S2 cells by using a dual-luciferase assay. LvJNK and Lvc-Jun were further confirmed to induce *IE1*, which is also named as *wsv069* (Figure 6A). Notably, another three *IEs*, including *wsv056*, *wsv249*, and *wsv403,* were also regulated by both LvJNK and Lvc-Jun *in vitro* (Figure 6A). A transcription-factor-binding motif search showed that there was at least one putative AP-1-binding motif present in the promoter regions of these *IEs* (Figure 6B). One AP-1-binding site, which was closest to the transcriptional start site (TSS), was chosen from *wsv056*, *wsv249,* and *wsv403* for EMSA assays to determine the potential interaction between these motifs and Lvc-Jun. The results showed that the rLvc-Jun protein could interact with these AP-1-binding motifs from *wsv056* (Figure 6C), *wsv249* (Figure 6D), and *wsv403* (Figure 6E). ChIP assays also demonstrated that Lvc-Jun could bind to the *wsv056*, *wsv249,* and *wsv403* promoters *in vivo* (Figure 6F). In summary, these results showed that the IE1-JNK-c-Jun loop was able to induce multiple WSSV *IE* genes, including *IE1* (*wsv069*), *wsv056*, *wsv249,* and *wsv403 in vivo* and *in vitro*.

## Discussion

Surface barriers provide the first line of defense to protect host from viral infection. Once the surface barriers have been breached, the invading virus is confronted by the innate immune system of the host (Troell et al., 2014). Although a variety of host defense mechanisms respond to these invasions, and in most cases effectively prevent invasive viral disease, increasing studies have shown that pathogens have co-evolved with their hosts and developed efficient strategies to circumvent and even hijack host defenses (Chemes et al., 2015, Hagai et al., 2014). Because the innate immune system has the critical role of controlling virus copies during the early stages of infection, the mechanisms employed by the invading virus to replicate itself within the host and evade the host immune defenses have attracted increasing interest. Crosstalk between the virus and host immune system is a hot topic for research regarding viral invasion of the host. MAPK pathways play a pivotal role in a wide range of cellular and physiological functions that positively impact viral replication, such as cell cycle control, cell survival, protein synthesis, and lipid metabolism (Arthur and Ley, 2013). Thus, it is not surprising that viruses hijack these pathways for their own benefit. However, even in well-studied insect (mosquito or *Drosophila*) and mammalian systems, little is known about the mechanism viruses used to manipulate the cellular MAPK signaling pathways. In this study, we clarified for the first time that the WSSV, a crustacean-infecting dsDNA virus, hijacks the host JNK pathway to enhance its replication by an IE1-driven positive feedback loop.

A common strategy that a virus uses to facilitate its infection or replication is to manipulate host cellular pathways (Hagai et al., 2014, Shah et al., 2018). Our finding that JNK activation by the WSSV facilitated virus replication in shrimps reflects a similar result in which virus-host interactions utilize the JNK pathway. Given the importance of MAPK signaling pathways in regulating immune responses, it is reasonable that some pathogens have developed mechanisms to directly modulate MAPK activation. The JNK signaling pathway in mammal or invertebrate hosts has been reported to be involved in or altered by infection with a variety of viruses. For example, the JNK pathway was activated by varicella zoster virus (VZV) infection and blockade of this pathway by SP600125-limited lytic replication and viral reactivation in human embryonic stem-cell-derived neurons (Kurapati et al., 2017). Further, JNK activation by the hepatitis B virus (HBV) X protein enhanced autophagosome formation, which was required for HBV replication in human HepG2 cells (Zhong et al., 2017). In the small brown planthopper (insect), JNK activation by the plant rice stripe virus (RSV) facilitated viral replication (Wang et al., 2017). In our present study, we demonstrated that disruption of the JNK pathway via RNAi or SP600125 inhibited WSSV replication in shrimp. Of note, the ability of the WSSV to hijack the JNK pathway might highlight a remarkably brilliant scheme by which the virus targets one of the evolutionarily earliest innate immune pathways, the MAPKs. The JNK pathway is a conserved immune pathway, and it presumably appeared very early in evolution before the emergence of multicellularity (Ausubel, 2005). In addition, JNK MAPKs have been regarded as essential genes, and lacking these genes can result in defects during development or morphogenesis (Sluss et al., 1996). Accordingly, JNK MAPKs appear to be driven by purifying selection, evolve more slowly, and thus have strong functional constraints (Li et al., 2011, Wilson et al., 1977). In contrast, viruses must continually evolve in order to colonize their hosts and take advantage of host cellular functions to guarantee their replication. Therefore, viruses have the potential to dominate in the host-virus arms race. However, the effectiveness of exploiting the evolutionarily conserved JNK MAPKs may bypass the need for the evolution of additional effectors or multiple strategies. In this regard, the WSSV could use a single effector protein (IE1) to effectively activate the JNK pathway by targeting the conserved JNK MAPK during infection. In addition, a wide range of viruses, including DNA and RNA viruses from plants, invertebrates, and vertebrates, have been shown to exploit this pathway, which suggests that targeting the JNK pathway, though by different means, could be an evolutionarily viral virulence strategy.

Cellular and biochemical experiments revealed that IE1 of the WSSV interacted with JNK via its JNK-binding motif, and in turn, promoted JNK autophosphorylation and substrate kinase activity. The autophosphorylation properties of MAPKs have been reported in vertebrates, *Arabidopsis,* and yeast but not invertebrates. A previous report demonstrated that human JNK2 and JNK3 underwent significant autophosphorylation and effectively phosphorylated their known substrate, ATF2, by *in vitro* phosphorylation experiments (Vogel et al., 2009). Another study demonstrated that only JNK2 isoforms, but not the JNK1 or JNK3 isoforms, have the ability to autophosphorylate and exhibit substrate kinase activity *in vitro* and *in vivo*, which does not require the participation of any upstream kinases (Cui et al., 2005). In addition, autophosphorylation of p38α has also been observed when it binds to the TAB1 protein, which does not require phosphorylation prior to p38 autoactivation (Thapa et al., 2018). Here, for the first time, an MAPK in an invertebrate possesses autophosphorylation properties as reported. Interestingly, IE1 of the WSSV utilized JNK autophosphorylation properties to achieve activation of the JNK pathway. This conclusion is strongly supported by phosphorylation assays in both the S2 cell line and *in vitro* phosphorylation system. Even though there are reports of other virus that hijack JNK pathway, our results demonstrated for the first time that a viral IE protein interacting with JNK directly and functioning as an enhancer of MAPK autophosphorylation established an IE1-JNK-c-Jun-IE1-positive feedback loop that represents a new mechanism of activation distinct from the well-known activation by MAPKK. Autoactivation of the JNK MAPK pathway facilitated by a viral protein could provide a novel strategy that other viruses might also use to exploit intracellular signaling pathways.

We also provide a possible explanation why activation of the JNK pathway facilitated WSSV replication. As mentioned above, IE1 of the WSSV was able to interact with JNK and promote autoactivation of JNK by autophosphorylation, and IE1 itself was also induced by the activation of the JNK-c-Jun cascade, which generated a positive feedback loop (Figure 6G). IE1 is a WSSV immediate-early protein, and its synthesis must be dependent on host transcription factors, which raises the question of how the JNK pathway is activated shortly after viral infection. It is well known that the JNK pathway is located at the crossroads between several pattern recognition receptor (PRR) signaling pathways, such as toll-like receptors (TLRs), RIG-I-like receptors (RLRs), NOD-like receptors (NLRs), C-type lectin receptors (CLRs), epidermal growth factor receptor (EGFR) signaling pathway, and ER stress signaling pathway (Arthur and Ley, 2013, Meng and Xia, 2011). Thus, WSSV infection (pathogenic stress) may activate one of these pathways. For example, a virus may induce ER stress that initially activates the JNK-c-Jun cascade. In addition, IE1 can be induced by other host transcription factors, including NF-κB, STAT, YY1, and HMGB, which also provides other means to transcriptionally activate *IE1* (Huang et al., 2017). The initial synthesis of IE1 can feedback to interact with JNK and promote its autophosphorylation, and in turn, trigger c-Jun to induce the transcription of several viral IE genes, including *IE1* (*wsv069*), *wsv056*, *wsv249,* and *wsv403* (Figure 6). These WSSV IE genes are highly implicated in viral replication and pathogenesis, and as such, silencing of *IE1* during WSSV infection led to a remarkable reduction in viral loads and an improved survival rate. Wsv056 has been able to stimulate G1/S transition by binding to the host retinoblastoma protein, which is deemed to be beneficial to virus genome replication (Ran et al., 2013). Wsv403 is also related to regulation of cell cycle progression, as it is able to interact with shrimp protein phosphatases (PPs) (Li et al., 2009). As such, this protein might be a regulator of both the initiation of primary infection and the reactivation of the WSSV in the host, as its transcription occurs during latency and increases when the lytic stage starts. Wsv249 encodes an E3-ligase, which contains an RING-H2 motif and interacts with a shrimp ubiquitin-conjugating enzyme to mediate ubiquitination (Li et al., 2009). Thus, it might regulate the function of host proteins by ubiquitination, thereby facilitating viral replication. Thus, the IE1-driven positive feedback loop can maintain sustained activation of the JNK-c-Jun cascade, which is able to boost the expression of several IE genes, as well as other potential viral genes regulated by this pathway that are beneficial to replication. Moreover, activation of the JNK-c-Jun cascade was still observed at 36 hpi, which is longer than a single viral replication cycle (approximately 20 h). However, it is unknown if this type of positive feedback loop can last for the entire infection period or if it is able to be interrupted by other mechanism. Of note, until now, no positive feedback loop has been reported where a DNA virus regulates viral replication via its viral proteins. To the best of our knowledge, we demonstrated for the first time that a DNA virus can form a viral protein-driven positive feedback loop to modulate activation of the MAPK pathway in shrimp. In addition, perhaps only a few IE genes are required to generate and maintain an efficient positive regulator loop. Further research into this viral mechanism could provide insights into how WSSV infection can cause 100% mortality in penaeid shrimps.

In summary, our results showed that the WSSV hijacks the host JNK pathway via IE1 targeting JNK to generate a positive feedback loop, which facilitated viral gene expression and replication. Our findings in this study provide insights for understanding the interplay between the WSSV and host and for the development of antiviral agents for the treatment of WSSV infection.

### Limitations of the Study

WSSV establishes the IE1/JNK/c-Jun positive feedback loop to drive replication observed in a shrimp *L. vannamei* model. Disruption of the IE1/JNK/c-Jun positive feedback loop is based on knockdown or inhibitor, without the inclusion of knockout experimental system. Our data show that IE1 can interact with LvJNK via its JBD motif, resulting in the enhanced LvJNK autophosphorylation, but the actual mechanism of IE1-mediated LvJNK autoactivation is still elusive.

## Methods

All methods can be found in the accompanying Transparent Methods supplemental file.

## Data and Code Availability

All reagents and experimental data are available within Transparent Methods or from corresponding author upon reasonable request.

## Supplemental Information

Document S1. Transparent Methods, Figures S1–S5, and Table S1
