# Supplementary material for: White Spot Syndrome Virus Establishes a Novel IE1/JNK/c-Jun Positive Feedback Loop to Drive Replication
Source: iScience. 2019 Nov 30;23(1):100752. doi: 10.1016/j.isci.2019.100752 (PMC6941876; doi:10.1016/j.isci.2019.100752)
Supplement: Document S1. Transparent Methods, Figures S1–S5, and Table S1 [file mmc1.pdf]

ISCI, Volume *23*

## Supplemental Information

# White Spot Syndrome Virus Establishes a Novel IE1/JNK/c-Jun Positive Feedback Loop to Drive Replication

**Sheng Wang, Haoyang Li, Shaoping Weng, Chaozheng Li, and Jianguo He**

Supplemental Information

## Supplemental Figures and Figure legends

**Figure S1.**

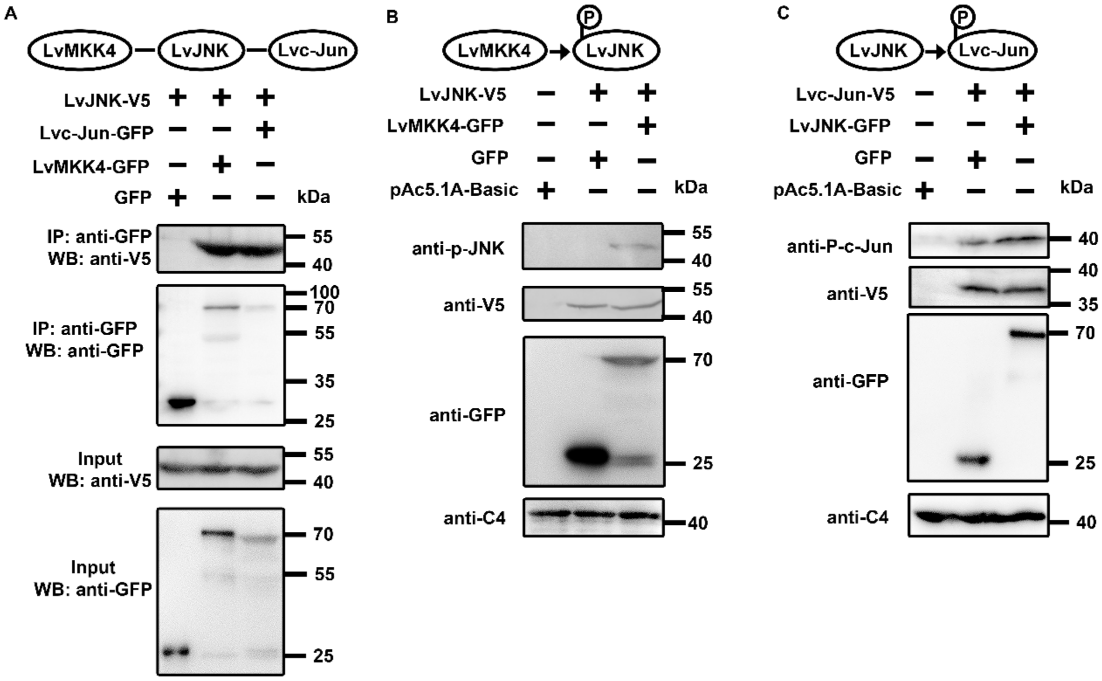


**Figure S1. The MKK4-JNK-c-Jun cascade was conserved in shrimp *L. vannamei*, related to Figure 1. (A)** The interaction of LvJNK with LvMKK4 or Lvc-Jun confirmed by Co-IP analysis. LvJNK-V5 was co-expressed in *Drosophila* S2 cells with Lvc-Jun-GFP and LvMKK4-GFP, respectively. The pAc5.1A-GFP was used as a negative control. GFP-tagged proteins were immunoprecipitated with an anti-GFP affinity gel, and then the immunoprecipitated complexes were analyzed by Western blotting with anti-V5 antibody and anti-GFP antibody. Five percent of the total cell lysate was also detected with the anti-V5 and anti-GFP antibodies as inputs. **(B–C)** The MKK4-JNK-c-Jun phosphorylation cascade was conserved *in vitro*. LvJNK-V5 was co-expressed with LvMKK4 in *Drosophila* S2 cells, and then the phosphorylation of LvJNK-V5 was probed with anti-p-JNK antibody (**B**). Lvc-Jun was phosphorylated by LvJNK in *Drosophila* S2 cells (**C**). Images were representative of three biological replicates (A–C).

**Figure S2.**

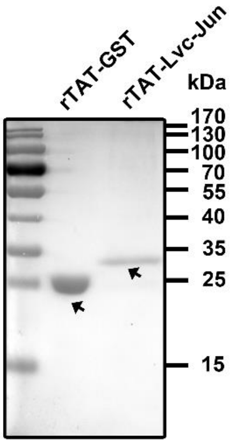


**Figure S2. Recombinant TAT-GST (rTAT-GST) and TAT-Lvc-Jun (rTAT-Lvc-Jun) were purified, related to Figure 2.** rTAT-GST and rTAT-Lvc-Jun were used for *in vivo* rescue experiments. The ORF of Lvc-Jun was cloned into the modified pGEX-rTAT for the recombinant proteins entering the shrimp cells. GST tag of rTAT-Lvc-Jun-GST was then removed using rPorcine Enterokinase from Glutathione Resin Kit. The purification productions of rTAT-GST and rTAT-Lvc-Jun were analyzed using SDS-PAGE and stained with Coomassie blue.

**Figure S3.**

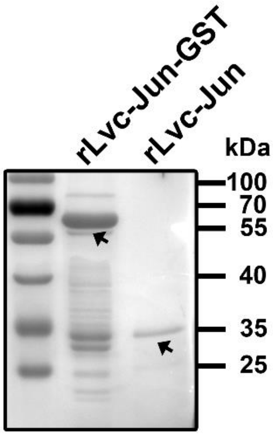


**Figure S3. Recombinant Lvc-Jun (rLvc-Jun) was obtained by removing the GST tag from rLvc-Jun-GST, related to Figure 3 and Figure 6.** The ORF of Lvc-Jun was cloned into the modified pGEX-4T plasmid to get rLvc-Jun-GST. GST tag of rLvc-Jun-GST was then removed using rPorcine Enterokinase from Glutathione Resin Kit. The purification productions of rLvc-Jun-GST and rLvc-Jun were analyzed using SDS-PAGE and stained with Coomassie blue, and rLvc-Jun was used for EMSA experiments.

**Figure S4.**

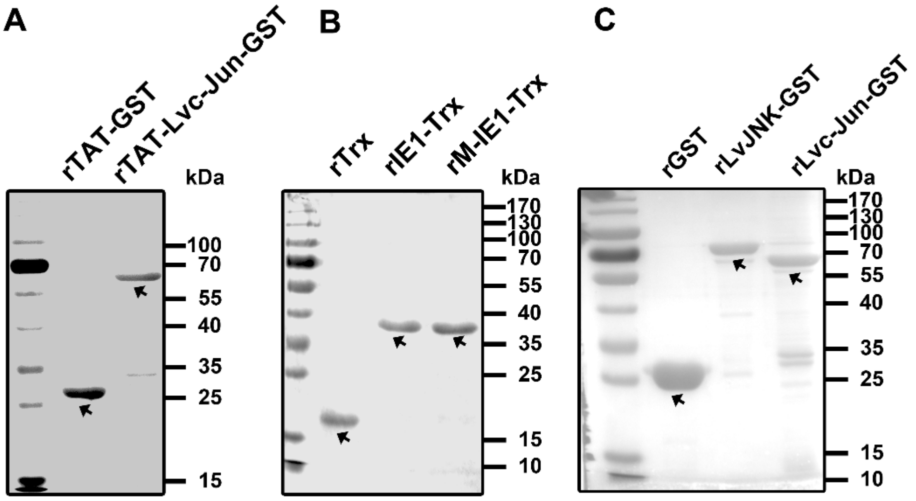


**Figure S4. Recombinant proteins were purified, related to Figure 2 and Figure 4. (A)** Purification of rTAT-GST and rTAT-Lvc-Jun-GST. These proteins were analyzed using SDS-PAGE and stained with Coomassie blue, and used for *in vivo* experiments. **(B)** IE1 and M-IE1 were constructed into pET-32a (+) vector to and transformed into *Escherichia coli* BL21 for the expression of rIE1-Trx, rM-IE1-Trx and rTrx. These proteins were analyzed using SDS-PAGE and stained with Coomassie blue and used for GST pulldown experiments. **(C)** Purification of rGST, rLvJNK-GST, and rLvc-Jun-GST. These proteins were analyzed using SDS-PAGE and stained with Coomassie blue and used for GST pulldown experiments.

**Figure S5.**

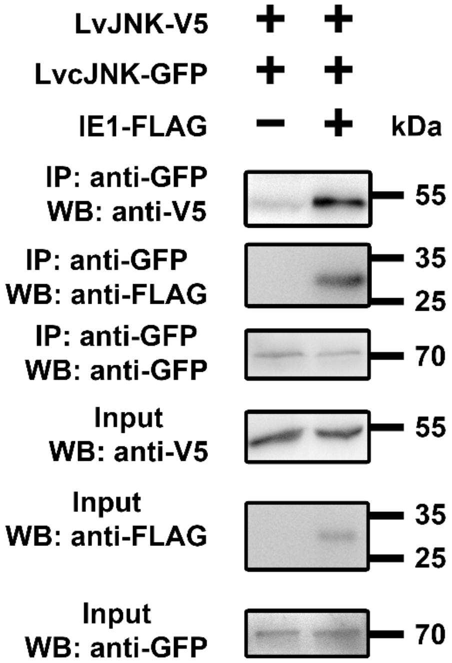


**Figure S5. Over expression of IE1 enhanced LvJNK-LvJNK combination, related to Figure 4.** The association of LvJNK-LvJNK with or without over-expression of IE1 was detected by Co-IP analysis. Five microgram of LvJNK-V5 plasmid and five microgram of LvJNK-GFP plasmid was co-transfected with or without five microgram of IE1-FLAG plasmid in

*Drosophila* S2 cells. Forty-eight hours later, cells were harvested. GFP-tagged proteins were immunoprecipitated with an anti-GFP affinity gel, and then the immunoprecipitated complexes were analyzed by Western blotting with anti-V5 antibody, anti-GFP antibody and anti-FLAG antibody. Five percent of the total cell lysate was also detected with the anti-V5 antibody, anti-GFP antibody and anti-FLAG antibody as inputs.

**Supplemental Table**

**Table S1. Summary of primers used in this paper, related to Figure 1 to Figure 6.**

| **Names** | **Sequences (5'–3')** |
|---|---|
| **Quantitative RT-PCR (qRT-PCR)** | |
| LvEF-1α-F | TATGCTCCTTTTGGACGTTTTGC |
| LvEF-1α-R | CCTTTTCTGCGGCCTTGGTAG |
| QLvMKK4-F | CCATATCCCAAGTGGAATTCTG |
| QLvMKK4-R | ATGCCACTAACAAACTCTGTC |
| QLvJNK-F | CCGCTACCCTGGCTATTCCTT |
| QLvJNK-R | TCGTGCTTGACTTGCTTTGAG |
| QLvc-Jun-F | GACGCCCTCCCAGTTCTTCTT |
| QLvc-Jun-R | CTGGTGGAGATGGCATCCTG |
| QIE1-F | GCACAACAACAGACCCTACCC |
| QIE1-R | GAAATACGACATAGCACCTCCAC |
| QVP28-F | AACACCTCCTCCTTCACCC |
| QVP28-R | GGTCTCAGTGCCAGAGTAGGT |
| **Semi-quantitative RT-PCR** | |
| Semi-IE1-F | ATTGAGGTGTTAAAGAAGCAGTTGT |
| Semi-IE1-R | ACAACAGCGATTACACCATTTCTAG |
| Semi-wsv056-F | CCCTTCATCTTCATCTCAAAAACTT |
| Semi-wsv056-R | TAGCAAGGAGCTCACAGTCTTATAT |
| Semi-wsv249-F | TAGGCGAGTCATGTTTCTTGCACCA |
| Semi-wsv249-R | CACCCAGATCGGCACAAAAAACACC |
| Semi-wsv403-F | GAGATTGAGTAAAATTTCTTGACGAT |
| Semi-wsv403-R | TACACACACAGAACCCACAAAAAC |
| **Absolute quantitative PCR (aq-PCR)** | |
| WSSV32678-F | TGTTTTCTGTATGTAATGCGTGTAGGT |
| WSSV32753-R | CCCACTCCATGGCCTTCA |
| TaqMan probe WSSV32706 | CAAGTACCCAGGCCCAGTGTCATACGTT |
| **Protein expression** | |
| IE1-HA-F | GGAATTCATGGCCTTTAATTTTGAAGACTC |
| IE1-HA-R | TTGGGCCCTACAAAGAATCCAGAAATCTCA |
| M-IE1-HA-F | GCAACAAACTGCTTGGCAGCATTCGCACAAGAGTTTATCAGCAACT |
| M-IE1-HA-R | AGTTGCTGATAAACTCTTGTGCGAATGCTGCCAAGCAGTTTGTTGC |

| | |
|---|---|
| LvJNK-GFP-F | GGGGTACCATGCCTCTTCTGAGTCCGCGGCC |
| LvJNK-GFP-R | TTGGGCCCTCTGTGGTTTGGGCGATGCTGGT |
| IE1-PET-F | GGAATTCATGGCCTTTAATTTTGAAGACTC |
| IE1-PET-R | AAGCTTTACAAAGAATCCAGAAATCTCATCA |
| Lvc-Jun-PGEX-F | CGGGATCCATGGAGGCAACCATGTACGAGGACG |
| Lvc-Jun-PGEX-R | CCGCTCGAGCTGGTGCGTTACGAAGGGGATC |
| LvJNK-PGEX-F | CGGAATTCATGCCTCTTCTGAGTCCGCGGC |
| LvJNK-PGEX-R | CCCTCGAGTCTGTGGTTTGGGCGATGCTGGTCC |
| **Dual-luciferase reporter assay** | |
| pIE(-128)-F | GGGGTACCTGAAAATGGCTGTTTGAATCATGTTAAG |
| pIE(-128)-R | GGAGATCTCTTGAGTGGAGAGAGAGAGCTAG |
| pIE(-102)-F | GGGGTACCAGGAATTTCCTTGTTACTCATTTAT |
| pIE(-102)-R | GGAGATCTCTTGAGTGGAGAGAGAGAGCTAG |
| pIE(-50)-F | GGGGTACCGGCGGAGCATATTTGTGTATATAAGAGCC |
| pIE(-50)-R | GGAGATCTCTTGAGTGGAGAGAGAGAGCTAG |
| **DsRNA templates amplification** | |
| dsRNA-LvMKK4-T7-F | GGATCCTAATACGACTCACTATAGGCAGCCTTGACTTCCCTGC CTC |
| dsRNA-LvMKK4-R | CGTTGGGTGAAAGACGTGGTG |
| dsRNA-LvMKK4-F | CAGCCTTGACTTCCCTGCCTC |
| dsRNA-LvMKK4-T7-R | GGATCCTAATACGACTCACTATAGGCGTTGGGTGAAAGACGTG GTG |
| dsRNA-LvJNK-T7-F | GGATCCTAATACGACTCACTATAGGGCTCATGAAACTCGTCAAT C |
| dsRNA-LvJNK-R | CCAGTTGCTCAATAATCTTA |
| dsRNA-LvJNK-F | GCTCATGAAACTCGTCAATC |
| dsRNA-LvJNK-T7-R | GGATCCTAATACGACTCACTATAGGCCAGTTGCTCAATAATCTT A |
| dsRNA-Lvc-Jun-T7-F | GGATCCTAATACGACTCACTATAGGACCATCCTCAAC AGCAACACG |
| dsRNA-Lvc-Jun-R | CGCTCCTGGCACTCCATATC |
| dsRNA-Lvc-Jun-F | ACCATCCTCAACAGCAACACG |
| dsRNA-Lvc-Jun-T7-R | GGATCCTAATACGACTCACTATAGGCGCTCCTGGCAC TCCATATC |
| dsRNA-IE1-T7-F | GGATCCTAATACGACTCACTATAGGCAATATGGACTTGACGGCT GG |
| dsRNA-IE1-R | GGCCTGGGTACTTGCACCTAC |
| dsRNA-IE1-F | CAATATGGACTTGACGGCTGG |
| dsRNA-IE1-T7-R | GGATCCTAATACGACTCACTATAGGGGCCTGGGTACTTGCACC TAC |
| dsRNA-GFP-T7-F | GGATCCTAATACGACTCACTATAGGCGACGTAAACGGCCACAAG TT |
| dsRNA-GFP-R | ATGGGGGTGTTCTGCTGGTAG |

| | |
|---|---|
| dsRNA-GFP-F | CGACGTAAACGGCCACAAGTT |
| dsRNA-GFP-T7-R | GGATCCTAATACGACTCACTATAGGATGGGGGTGTTCTGCTGGTAG |
| **EMSA** | |
| Bio-IE1-AP-1-1-F | GAAAATGGCTGTTTGAATCATGTTAAGGAATT |
| Bio-IE1-AP-1-1-R | AATTCCTTAACATGATTCAAACAGCCATTTTC |
| unBio-IE1-AP-1-1-F | GAAAATGGCTGTTTGAATCATGTTAAGGAATT |
| unBio-IE1-AP-1-1-R | AATTCCTTAACATGATTCAAACAGCCATTTTC |
| Mut-unBio-IE1-AP-1-1-F | GAAAATCGCTGTTCCCATCCTGTTAAGGAATT |
| Mut-unBio-IE1-AP-1-1-R | AATTCCTTAACAGGATGGGAACAGCGATTTTC |
| Bio-IE1-AP-1-2-F | GGAATTTCCTTGTTACTCATTTATTCCTAGA |
| Bio-IE1-AP-1-2-R | TCTAGGAATAAATGAGTAACAAGGAAATTCC |
| unBio-IE1-AP-1-2-F | GGAATTTCCTTGTTACTCATTTATTCCTAGA |
| unBio-IE1-AP-1-2-R | TCTAGGAATAAATGAGTAACAAGGAAATTCC |
| Mut-unBio-IE1-AP-1-2-F | GGAATTTCCTTGTTACGGGTTTATTCCTAGA |
| Mut-unBio-IE1-AP-1-2-R | TCTAGGAATAAACCCGTAACAAGGAAATTCC |
| Bio-056-AP-1-F | GTACCAGATGTGAGTCAAACCGTTTCTGG |
| Bio-056-AP-1-R | CCAGAAACGGTTTGACTCACATCTGGTAC |
| unBio-056-AP-1-F | GTACCAGATGTGAGTCAAACCGTTTCTGG |
| unBio-056-AP-1-R | CCAGAAACGGTTTGACTCACATCTGGTAC |
| Mut-unBio-056-AP-1-F | GTACCAGATGTGAGGGGAACCGTTTCTGG |
| Mut-unBio-056-AP-1-R | CCAGAAACGGTTCCCCTCACATCTGGTAC |
| Bio-249-AP-1-F | TAGGAATTTCGCTGACGTCAATAAACTGGTTT |
| Bio-249-AP-1-R | AAACCAGTTTATTGACGTCAGCGAAATTCCTA |
| unBio-249-AP-1-F | TAGGAATTTCGCTGACGTCAATAAACTGGTTT |
| unBio-249-AP-1-R | AAACCAGTTTATTGACGTCAGCGAAATTCCTA |
| Mut-unBio249-AP-1-F | TAGGAATTTCGCACGCGTCGATAAACTGGTTT |
| Mut-unBio-249-AP-1-R | AAACCAGTTTATCGACGCGTGCGAAATTCCTA |
| Bio-403-AP-1-F | ATTTCCAAGAATTTTGCTGACGTCAATGGACC |
| Bio-403-AP-1-R | GGTCCATTGACGTCAGCAAAATTCTTGGAAAT |
| unBio-403-AP-1-F | ATTTCCAAGAATTTTGCTGACGTCAATGGACC |
| unBio-403-AP-1-R | GGTCCATTGACGTCAGCAAAATTCTTGGAAAT |
| Mut-unBio-403-AP-1-F | ATTTCCAAGAATTTTGCACGCGTCGTGGACC |
| Mut-unBio-403-AP-1-R | GGTCCACGACGCGTGCAAAATTCTTGGAAAT |

## Transparent Methods

### Animals

Healthy shrimp (*L. vannamei*) with an average weight of approximately 5 *g* each were purchased from the local shrimp farm in Zhuhai, Guangdong Province, China. The shrimp were acclimated in aerated artificial seawater (2.5% salinity) for two days prior to performing any experiments. Shrimp were fed daily with a commercial shrimp diet (HAID Group, Guangzhou, Guangdong, China).

**Preparation of WSSV inoculums, shrimp challenge, and sampling**

The WSSV inoculum was prepared as previously described (Li et al., 2018). In brief, the WSSV (a Chinese isolate, AF332093) was isolated from WSSV-infected shrimp muscle tissue that was stored at -80 °C. Those muscle tissues were homogenized, and the viral titers were determined by absolute q-PCR as previously described (Qiu et al., 2014). Each shrimp in WSSV-treated groups received an intraperitoneal injection of 1 × $10^5$ copies of extracted WSSV DNA in 50 µl PBS solution (pH 7.4) at the second abdominal segment with a 1-ml syringe (Becton Dickinson, Franklin Lakes, NJ, USA; cat. no. 7276801). For qRT-PCR, hemocytes from 9 WSSV-challenged shrimp (3 shrimp in each sample, 3 total samples) were sampled at various time points to extract RNA for cDNA synthesis.

For Western blotting, hemocytes from 30 WSSV-challenged shrimp (10 shrimp in each sample, 3 total samples) at 0, 8, 36 hours post WSSV infection were harvested and then lysed in IP lysis buffer (Pierce, Appleton, WI, USA; cat. no. 87788) with a protease and phosphatase inhibitor cocktail (Merck, Kenilworth, NJ, USA; cat. no. 524628), and then centrifuged at 12,000 × *g* for 10 minutes at 4 ºC to remove cell debris. Then 5× loading buffer was added, and the samples were boiled for 10 minutes.

**RNA extraction, genomic DNA extraction, and cDNA synthesis**

Total RNA was isolated using Trizol reagent (Thermo Fisher Scientific, Waltham, MA, USA; cat. no. 15596026), and then dissolved in 50 µl of RNase-free water (Takara, Kusatsu, Shiga 525-0058, Japan; cat. no. 9012). The genomic DNA was extracted using TIANGEN Marine Animal DNA Kit (TIANGEN, Beijing, China; cat. no. GD3311-02), according to the manufacturer's instructions. Total RNA (1 µg) was used in a 20 µl reverse transcription reaction. TransScript One-Step gDNA Removal and cDNA Synthesis SuperMix (TransGen Biotech, Beijing, China; cat. no. AT311-02) was used for the synthesis of first-strand cDNA.

**qRT-PCR**

qRT-PCR assays were performed to assess the mRNA levels in the pathogenic challenge experiments or the *in vivo* RNAi experiments. Expression levels of *LvMKK4*, *LvJNK*, *Lvc-Jun*, *IE1,* and *VP28* were detected using LightCycler 480 System (Roche, Basel, Germany) in a final reaction volume of 10 µl, which was comprised of 1 µl of 1:10 cDNA diluted with $ddH_2O$, 5 µl of GoTaq qPCR Master Mix (Promega, Madison, WI, USA; cat. no. A6002), and 250 nM of specific primers (Table S1). The cycling program was as follows: 1 cycle of 95 °C for 2 minutes, followed by 40 cycles of 95 °C for 15 s, 62 °C for 1 minute, and 70 °C for 1 s. Cycling ended at 95 °C with a 5 °C/s calefactive velocity to create the melting curve. The expression level of each gene was calculated using the Livak ($2^{-\triangle\triangle CT}$) method after normalization to *EF-1a* (GenBank accession No. GU136229). Primers are listed in Table S1.

**Knockdown of specific genes by dsRNA-mediated RNAi**

T7 RiboMAX Express RNAi System kit (Promega; cat. no. P1700) was used to generate dsRNA-LvMKK4, dsRNA-LvJNK, dsRNA-Lvc-Jun, dsRNA-IE1, and dsRNA-GFP with primers containing a 5' T7 RNA polymerase binding site (Table S1). The dsRNA quality was checked after annealing by gel electrophoresis. Each shrimp received an intraperitoneal injection at the second abdominal segment of dsRNAs (2 µg/ g shrimp in 50 µl of PBS) or equivalent PBS. Hemocytes were collected from shrimp 48 hours after the dsRNA injection, and total RNA was extracted and assessed by qRT-PCR using the corresponding primers to evaluate the efficacy of RNAi.

**Survival experiments**

To knockdown specific host gene expression, healthy shrimp received an intramuscular injection of 10 μg of dsRNA or PBS only. Forty-eight hours later, shrimp were injected again with $1 \times 10^5$ copies of WSSV DNA, and mock-challenged with PBS as a control ($n$ = 30).

To suppress WSSV IE1 expression, dsRNA-IE1 injection was performed at the same time as the WSSV challenge ($n$ = 40).

For the r-Lvc-Jun rescue experiment, 10 μg of TAT-tag recombinant proteins were co-injected with 10 μg of dsRNA per shrimp, and the WSSV inoculum was injected into shrimp 48 hours later as the secondary injection ($n$ = 30).

For the inhibitor SP600125 treated experiment, we dissolved JNK specific inhibitor, SP600125 (CST; cat. no. 8177S) in DMSO to reach a concentration of 50 mM. Each shrimp received 50 μl JNK-inhibitor mixtures (6.8 μl SP600125+ 43.2 μl PBS) or 50 μl DMSO mixures (6.8 μl DMSO+ 43.2 μl PBS). And the WSSV inoculum was injected into shrimps 4 hours later as the secondary injection ($n$ = 30).

The mortality of each group was recorded every four hours until 6 days post-WSSV infection. The log-rank test method (GraphPad Prism software, GraphPad, San Diego, CA, USA) was used to analyze the differences between groups.

**Absolute q-PCR**

Absolute q-PCR was performed to monitor viral titers in shrimp. Briefly, we collected gills from shrimp 48 hours post-WSSV infection ($n$ = 11 in dsRNA-*IE1* experiment; $n$ = 8 in knockdown and rescue experiments). Gill DNA was extracted as described above. The concentration of WSSV genome copies was measured by absolute q-PCR using WSSV32678-F and WSSV32753-R primers (Table S1) and a TaqMan fluorogenic probe as described previously (Qiu et al., 2014). The WSSV genome copy number in 1 μg of shrimp DNA was then calculated.

**Plasmids construction**

An artificial pAc5.1/3×HA vector was generated by replacing the C-terminal V5-His tag with a 3 × HA tag from the pAc5.1/V5-His A vector (Thermo Fisher Scientific). And the artificial pAc5.1/FLAG vector was generated by replacing the C-terminal V5-His tag with a FLAG tag from the pAc5.1/V5-His A vector. The open reading frame (ORF) of *IE1* was amplified and cloned into the pAc5.1/3×HA vector and pAc5.1/FLAG to express an HA-tagged IE1 protein and FLAG-tagged IE1 protein, respectively. LvJNK-GFP was obtained by cloning the ORF of *LvJNK* without a stop codon into the pAc5.1A-GFP vector (Li et al., 2012). The M-IE1-HA plasmid with a mutated JNK binding motif was obtained by using overlap extension PCR. Briefly, two overlapping DNA fragments were amplified from IE1-HA using the primer pairs IE1-HA-F/M-IE1-HA-R and M-IE1-HA-F/IE1-HA-R. A single product was obtained by PCR using the two separate DNA fragments pooled together as templates and the IE1-HA-F/IE1-HA-R primer pair (Table S1). The final PCR product was subcloned into the pAc5.1/3×HA vector to express the HA-tagged M-IE1 protein.

The reporter gene vectors, including pIE (-128) (full length *IE1* promoter), pIE (-102) (*IE1* promoter with the first AP-1 binding motif deleted), and pIE (-50) (*IE1* promoter with two AP-1 binding motifs deleted) were constructed in the pGL3-Basic vector using the primers listed in Table S1.

Protein expression plasmids, including pAc-Lvc-Jun-GFP, pAc-Lvc-Jun-V5,

pAc-LvJNK-V5, pAc-LvMKK4-GFP, and reporter genes vectors, including pGL3-*wsv051*, pGL3-*wsv056*, pGL3-*wsv078*, pGL3-*wsv079*, pGL3-*wsv080*, pGL3-*wsv083*, pGL3-*wsv091*, pGL3-*wsv100*, pGL3-*wsv101*, pGL3-*wsv103*, pGL3-*wsv108*, pGL3-*wsv078*, pGL3-*wsv087*, pGL3-*wsv249*, pGL3-*wsv358*, pGL3-*wsv403*, pGL3-*wsv465*, and pRL-TK, were obtained from our previous studies (Li et al., 2015; Li et al., 2016; Wang et al., 2016; Wang et al., 2018).

**Co-immunoprecipitation**

Co-immunoprecipitation (CoIP) assays *in vitro* were performed to confirm interaction between proteins. In brief, 48 hours after transfection, *Drosophila* S2 cells were harvested and washed with ice-cold PBS three times and then lysed in IP lysis buffer with Halt Protease Inhibitor Cocktail (Thermo Fisher Scientific; cat. no. 87786). The supernatants (100 µl) were incubated with 30 µl of agarose affinity gel with anti-GFP (MBL International Corporation, Lexington Avenue, NY, USA; cat. no. D153-8) or anti-HA affinity gel (Sigma-Aldrich, St. Louis, MO, US; cat. no. A2095) at 4 ºC for four hours. The agarose affinity gels were washed with PBS five times and subjected to SDS-PAGE assay. Five percent of each total cell lysate was also examined as the input control.

Co-IP assays *in vivo* were performed to confirm interactions between endogenous proteins in shrimp. Proteins from shrimp hemocytes were extracted with IP lysis buffer and incubated with a normal rabbit IgG antibody (Cell Signaling Technology (CST), Danvers, MA, USA; cat. no. 2729S) or anti-IE1 antibody (Genecreate, Wuhan, China) for 3 hours at 4 ºC. The mixture was then incubated with protein G agarose beads (CST; cat. no. 37478S) for 3 hours at 4 ºC, and then the pellet was washed with PBS five times. The resulting pellet (bound protein, antibody, and protein G) was analyzed by Western blotting. For detection of endogenous LvMKK4-LvJNK-Lvc-Jun interaction induced by WSSV infection, hemocytes were harvested at 0, 8, and 36 hpi, and anti-JNK antibody (CST; cat. no. 9252L) was used. Five percent of the cell lysate was loaded as the input control.

**Phosphorylation assays *in vitro***

To explore whether LvJNK could be phosphorylated by LvMKK4, LvJNK-V5 was co-transfected with LvMKK4-GFP or pAc5.1-GFP (as a control) into *Drosophila* S2 cells. Forty-eight hours post-transfection, S2 cells were harvested, lysed in IP lysis buffer with protease and phosphatase inhibitors, and detected via western blot analysis. The effects of IE1 on the phosphorylation states of LvJNK, as well as Lvc-Jun, were also detected in S2 cells.

To explore whether LvJNK could undergo autophosphorylation, phosphorylation of LvJNK proteins cloned with a GST tag (LvJNK-GST) was detected in a phosphorylation system *in vitro*. In brief, the purified LvJNK-GST proteins and ATP (CST; cat. no. 9804S) were added into 10 × Kinase buffer (CST; cat. no. 9802S) to obtain a 1 × reaction system. In the control, the ATP was replaced by $ddH_2O$. The mixtures were incubated for 1 hour at 30 ºC on MIX Vertical (Huierbio, Luoyang, China; HR-13) and then boiled with 5 × loading buffer (GenScript, Piscataway, NJ, USA; cat. no. MB01015) for western blot analysis and Coomassie blue staining. To investigate the effects of IE1 on LvJNK autophosphorylation, we probed the phosphorylation levels of LvJNK in the presence of IE1-Trx or Trx (as a control) as described above.

**Western blot**

Protein samples were separated in SDS-PAGE gels, transferred to PVDF membranes (GE Healthcare, Chicago, IL, USA), and incubated with the appropriate antibodies. The

primary antibodies used in Western blotting included a rabbit anti-GFP antibody (Sigma-Aldrich; cat. no. G1544-100UL), rabbit anti-V5 antibody (Merck Millipore, Burlington, MA, USA; cat. no. AB3792), rabbit anti-HA antibody (Sigma-Aldrich; cat. no. H6908-100UL), rabbit anti-FLAG antibody (Sigma-Aldrich; cat. no. F7425), rabbit anti-phosphorylation MKK4 antibody (CST; cat. no. 9156S), rabbit anti-MKK4 antibody (CST; cat. no. 9152S), rabbit anti-JNK antibody (CST; cat. no. 9252S), rabbit anti-phosphorylation JNK antibody (CST; cat. no. 9521S), rabbit anti-phosphorylation c-Jun antibody (CST; cat. no. 3270S), rabbit anti-c-Jun antibody (CST; cat. no. 9165S), and mouse anti-actin clone C4 antibody (Merck Millipore; cat. no. MAB1501). The secondary antibodies used were an anti-mouse IgG HRP-conjugate (Promega; cat. no. W402B) and anti-rabbit IgG HRP-conjugate (Promega; cat. no. W401B). Both primary and secondary antibodies were incubated in TBS-T with 0.5% BSA. Membranes were developed with the enhanced chemiluminescent (ECL) blotting substrate (Thermo Scientific) and chemiluminescence was detected using the 5200 Chemiluminescence Imaging System (Tanon). For relative densitometry of phosphorylation levels of LvMKK4, LvJNK and Lvc-Jun proteins, the immunoblotted protein bands density were analyzed using the ImageJ software 1.6.0 (National Institutes of Health, Bethesda, MD) and calculated one by one. Phosphorylation protein band density was then normalized to the corresponding total protein density in the lanes to get the ratio of p-MKK4/MKK4, p-JNK/JNK and p-Jun/Jun. Statistical analysis of densitometry data from three independent experiments was performed by using the Student's t test.

**Dual luciferase reporter assay**

To detect the activation of *IE1* promoter by Lvc-Jun, *Drosophila* S2 cells were cultured in a 24-well plate for 24 hours. The cells in each well were then transfected with 0.2 µg of firefly luciferase reporter-gene plasmids, 0.04 µg of pRL-TK renilla luciferase plasmid (internal control; Promega; cat. no. E2241), and 0.1 µg/ 0.3 µg/ 0.5 µg protein expression plasmids or 0.5 µg pAc5.1-V5 plasmid (as control). Forty-eight hours post-transfection, the cells were harvested, lysed, and then 60% of the lysate was used to measure the induction of the reporter genes via Dual-Glo Luciferase Assay System (Promega; cat. no. E2920). The remaining 40% of the S2 cell lysate was analyzed via western blot to detect the expression levels of proteins. All of the experiments were repeated three times. To screening all 21 IE genes induction by JNK pathway, *Drosophila* S2 cells were cultured in a 96-well plate for 24 hours and then transfected with 0.05 µg of firefly luciferase reporter-gene plasmids, 0.01 µg of pRL-TK renilla luciferase plasmid, and 0.05 µg protein expression plasmids or 0.05 µg pAc5.1-V5 plasmid (as control). Forty-eight hours post-transfection, the cells were harvested, lysed, and then measured via Dual-Glo Luciferase Assay System.

**Immunofluorescence and confocal laser scanning microscopy**

The hemocytes from WSSV-injected shrimp at 0, 8, 12 and 36 hpi were centrifuged at 3000 × *g* for 10 minutes at 4 ºC. The cells were washed twice with PBS and spread onto slides. After 30 minutes, remove PBS and fixed cells in 4% paraformaldehyde diluted in PBS at 25 ºC for 15 minutes. The cells were then permeabilized with methanol at -20 ºC for 10 minutes. After washing slides for three times, the hemocytes were blocked with 3% bovine serum albumin (diluted in PBS) for 1 hour at 25 ºC and then incubated with a mixture of primary antibodies (1:100, diluted in blocking reagent) overnight (about 8 hours) at 4 ºC. The primary antibodies used in immunofluorescence (IF) were rabbit anti-c-Jun antibody (CST; cat. no. 9525S),

mouse anti-β-actin antibody (Sigma-Aldrich; cat. no. A2228), mouse anti-IE1 antibody (Genecreate), and rabbit anti-JNK antibody (Abcam; cat. no. 179461), depending on the experiment. The slides were washed with PBS six times and then incubated with 1:1000 diluted anti-rabbit IgG (H+L), F (ab')2 fragment (Alexa Fluor 488 Conjugate; CST; cat. no. 4412S), and anti-mouse IgG (H+L), F (ab')2 Fragment (Alexa Fluor 594 Conjugate; CST; cat. no. 8890S) for 1 hour at 25 ºC. The cell nuclei were stained with 4-6-diamidino-2-phenylindole (DAPI) (Beyotime, Shanghai, China; cat. no. C1002) for 10 minutes. Finally, the slides were observed with a confocal microscope (Leica, Wetzlar, Germany ; TCS-SP5) after washing six times with PBS.

**Recombinant protein expression and purification**

The fragments encoding the wide-type IE1 and the M-IE1 with the JNK binding domain mutated were acquired by PCR amplification and ligated into the pET-32a (+) vector (GE Healthcare, Chicago, IL, USA). The full-length ORFs of Lvc-Jun and LvJNK were also acquired by PCR and ligated into pGEX-4T. To facilitate the cellular uptake of recombinant proteins, a cell-penetrating TAT peptide (TATGGCAGGAAGAAGCGGAGACAGCGACGAAGA) was fused to the Glutathione-S-transferase (GST) tag of the pGEX-4T-1 vector using the ClonExpress II One Step Cloning Kit (Vazyme, Nanjing, China; cat. no. C112-01) (Li et al., 2017). Recombinant Lvc-Jun was cloned into the modified pGEX-rTAT for the recombinant proteins entering the shrimp cells (Fig. S4A). The recombinant plasmids IE1-pET-32a, M-IE1-pET-32a, pET-32a, rTAT-Lvc-Jun-pGEX, rTAT-pGEX, Lvc-Jun-pGEX, pGEX, and LvJNK-pGEX were transformed into *Escherichia coli* BL21 for the expression of rIE1-Trx, rM-IE1-Trx, rTrx, rTAT-Lvc-Jun-GST, rLvc-Jun-GST, rGST, rTAT-GST, and rLvJNK-GST proteins, respectively. The transformed bacteria were cultured in Luria-Bertani medium (Sigma-Aldrich; cat. no. L3022) in the presence of 500 µg/ ml ampicillin (Beyotime; cat. no. ST007) at 37 ºC. When the density of the cultures reached an OD600 of 0.8, the proteins rTrx, rIE1-Trx, and rM-IE1-Trx were induced with 0.1 mM isopropyl-β-D-thiogalactopyranoside (IPTG) (Beyotime; cat. no. ST097) at 16 ºC overnight. Purification of Trx-tagged proteins was performed with a Ni-NTA Agarose (Thermo Fisher Scientific; cat. no. 30210), according to the manufacturer’s instructions. The purified Trx-tagged proteins were dialyzed in PBS overnight and then checked by SDS-PAGE followed by Coomassie blue staining (Fig. S4B). The other GST-tagged proteins were induced with 1 mM IPTG at 37 ºC for 4 hours. Purification of GST-tagged proteins was performed with a Glutathione Resin Kit (Genescript; cat. no. L00208) according to the manufacturer’s instructions. The purified rGST, rLvJNK-GST, and rLvc-Jun-GST proteins were confirmed by SDS-PAGE followed by Coomassie blue staining (Fig. S4C). Recombinant rTAT-Lvc-Jun and rLvc-Jun proteins were obtained from rTAT-Lvc-Jun-GST and rLvc-Jun-GST proteins by removing the GST-tag using rPorcine Enterokinase from Glutathione Resin Kit (Genescript; cat. no. L00208), respectively. Primers used are listed in Table S1.

**Electrophoretic mobility shift assay (EMSA)**

The biotinylated or unbiotinylated oligonucleotides of the putative AP-1 binding motifs and unbiotinylated oligonucleotides with mutated AP-1 binding motifs were designed and are listed in Table S1. All of the probes were synthesized by Life Technologies (Carlsbad, CA, USA). The synthesized oligonucleotides were diluted to 10 µM and then annealed to double-stranded probes. For a 20-µl incubation system, 20 fmol of unmutated-bio-probes and 2 µg of purified

rLvc-Jun or rGST were used in each sample. In competition binding assays, the complexes of wild-type probes and proteins were challenged with the unbiotinylated probes (or M-unbio probes) at 10-fold, 50-fold, or 100-fold molar excess over the labeled probes. The EMSA was performed according to the manufacturer's instructions from LightShift Chemiluminescent EMSA Kit (Thermo Fisher Scientific; cat. no. 20148).

**Primary hemocyte culture, SP600125, and TPA treatment**

Hemocytes from healthy shrimp *L. vannamei* were collected, suspended in serum-free Leibovitz-15 (L15; Sigma-Aldrich) growth medium, seeded in 25 $cm^2$ bottles, and maintained at 27 ºC. After a 30-minute incubation, fresh L15 with 15% FBS was added. For the inhibitor-treated groups, cells were treated with 10 μM of a JNK specific inhibitor, SP600125 (CST; cat. no. 8177S). For the TPA-treated groups, cells were treated with TPA (1 μM) (CST; cat. no. 4174S), while the control groups were treated with DMSO. After 1 hour, all of the groups were then infected with WSSV virions at a final concentrate of $1 \times 10^7$ copies/ml. Hemocytes were harvested at 0 and 8 hours post-infection.

**Chromatin immunoprecipitation assay (ChIP)**

Hemocytes from shrimp that were infected with WSSV for 24 hours were used for the ChIP assays. ChIP assays were performed according to the manufacturer’s instructions for the SimpleChIP Enzymatic Chromatin IP Kit (Magnetic Beads; CST; cat. no. 9003) with 6-tube Magnetic (CST; cat. no. 7017S). In brief, five percent of hemocytes were transferred into a new tube and stored for input detection. Other hemocytes were cross-linked first. And then nuclei preparation, chromatin digestion, and chromatin digestion analysis were performed according to the manufacturer’s instructions. A 10-μl aliquot of the diluted chromatin sample was transferred to a microcentrifuge tube as ChIP input. For the two immunoprecipitation reactions, 500 μl of the diluted chromatin was transferred to 1.5 ml microcentrifuge tubes and 1 μg of rabbit c-Jun antibody (CST; cat. no. 9165S) and 1 μg of normal rabbit IgG antibody (provided by the kit) was added, respectively. The IP samples were incubated for 4 hours to overnight at 4 °C with rotation. Then 30 μl of Protein G Magnetic Beads (provided by the kit) were added to each IP reaction and incubated for 2 hours at 4 °C with rotation. The supernatant was removed after placing the tubes in the magnetic separation rack. The pelleted protein G magnetic beads were washed with low salt buffer three times. Twenty percent of pelleted protein G magnetic beads were loaded with Loading Buffer and stored for ChIP bait protein detection. Then 1 ml of a high salt buffer was added to the other beads and incubated at 4 °C for 5 minutes with rotation. The supernatant was removed. The chromatin was eluted from the antibody/protein G magnetic bead complexes, and the cross-links were reversed. The DNA was purified using a spin column. The resulting purified DNA was subjected to semi-quantitative RT-PCR with 21–25 cycles of amplification. Primers were designed to amplify the promoters of *wsv056*, *wsv249*, *wsv403*, and *IE1* (Table S1). The PCR products were analyzed using agarose gel electrophoresis.

**Pull-down**

We incubated 200 μl of rJNK-GST with 200 μl of Trx-tagged protein (rIE1-Trx, rM-IE1-Trx, or rTrx) solutions (1 μg/ μl, diluted in PBS) at 4 ºC for 30 minutes. Then 20 μl of GST-bind resin was added to each incubation system and incubated at 4 ºC for 2 hours. The resin was washed with PBS thoroughly and then analyzed using SDS-PAGE and Coomassie blue staining.

### Statistical analysis

All of the data are presented as mean ± SD. Student's *t* test was used to calculate the comparisons between groups of numerical data. For survival rates, data were subjected to statistical analysis using GraphPad Prism software to generate the Kaplan ± Meier plot (log-rank $\chi^2$ test). The following *P* values were considered to be statistically significant: $*P < 0.05$ and $**P < 0.01$.

## Supplemental References


Li, C., Chen, Y.X., Zhang, S., Lu, L., Chen, Y.H., Chai, J., Weng, S., Chen, Y.G., He, J., Xu, X., 2012. Identification, characterization, and function analysis of the Cactus gene from *Litopenaeus vannamei*. PLoS One 7, e49711.

Li, C., Li, H., Wang, S., Song, X., Zhang, Z., Qian, Z., Zuo, H., Xu, X., Weng, S., He, J., 2015. The c-Fos and c-Jun from *Litopenaeus vannamei* play opposite roles in Vibrio parahaemolyticus and white spot syndrome virus infection. Dev Comp Immunol 52, 26-36.

Li, H., Wang, S., Lu, K., Yin, B., Xiao, B., Li, S., He, J., Li, C., 2017. An invertebrate STING from shrimp activates an innate immune defense against bacterial infection. FEBS Lett. 591, 1010-1017.

Li, H., Wang, S., Qian, Z., Wu, Z., Lu, K., Weng, S., He, J., Li, C., 2016. MKK6 from pacific white shrimp *Litopenaeus vannamei* is responsive to bacterial and WSSV infection. Mol Immunol 70, 72-83.

Li, H., Yin, B., Wang, S., Fu, Q., Xiao, B., Lu, K., He, J., Li, C., 2018. RNAi screening identifies a new Toll from shrimp *Litopenaeus vannamei* that restricts WSSV infection through activating Dorsal to induce antimicrobial peptides. PLoS Pathog 14, e1007109.

Qiu, W., Zhang, S., Chen, Y.G., Wang, P.H., Xu, X.P., Li, C.Z., Chen, Y.H., Fan, W.Z., Yan, H., Weng, S.P., FrancisChan, S., He, J.G., 2014. *Litopenaeus vannamei* NF-kappaB is required for WSSV replication. Dev Comp Immunol 45, 156-162.

Wang, S., Qian, Z., Li, H., L, K., Xu, X., Weng, S., He, J., Li, C., 2016. Identification and characterization of MKK7 as an upstream activator of JNK in *Litopenaeus vannamei*. Fish Shellfish Immunol 48, 285-294.

Wang, S., Yin, B., Li, H., Xiao, B., Lu, K., Feng, C., He, J., Li, C., 2018. MKK4 from *Litopenaeus vannamei* is a regulator of p38 MAPK kinase and involved in anti-bacterial response. Dev Comp Immunol 78, 61-70.
